# A versatile optimization framework for porous electrode design†

**DOI:** 10.1039/d3dd00247k

**Published:** 2024-04-25

**Authors:** Maxime van der Heijden, Gabor Szendrei, Victor de Haas, Antoni Forner-Cuenca

**Affiliations:** a Electrochemical Materials and Systems, Department of Chemical Engineering and Chemistry, Eindhoven University of Technology PO Box 513 5600 MB Eindhoven Netherlands a.forner.cuenca@tue.nl m.v.d.heijden@tue.nl szendrei.gabor09@gmail.com v.d.haas@student.tue.nl

## Abstract

Porous electrodes are performance-defining components in electrochemical devices, such as redox flow batteries, as they govern the electrochemical performance and pumping demands of the reactor. Yet, conventional porous electrodes used in redox flow batteries are not tailored to sustain convection-enhanced electrochemical reactions. Thus, there is a need for electrode optimization to enhance the system performance. In this work, we present an optimization framework to carry out the bottom-up design of porous electrodes by coupling a genetic algorithm with a pore network modeling framework. We introduce geometrical versatility by adding a pore merging and splitting function, study the impact of various optimization parameters, geometrical definitions, and objective functions, and incorporate conventional electrode and flow field designs. Moreover, we show the need for optimizing geometries for specific reactor architectures and operating conditions to design next-generation electrodes, by analyzing the genetic algorithm optimization for initial starting geometries with diverse morphologies (cubic and a tomography-extracted commercial electrode), flow field designs (flow-through and interdigitated), and redox chemistries (VO^2+^/VO_2_^+^ and TEMPO/TEMPO^+^). We found that for kinetically sluggish electrolytes with high ionic conductivity, electrodes with numerous small pores and high internal surface area provide enhanced performance, whereas for kinetically facile electrolytes with low ionic conductivity, low through-plane tortuosity and high hydraulic conductance are desired. The computational tool developed in this work can further expanded to the design of high-performance electrode materials for a broad range of operating conditions, electrolyte chemistries, reactor designs, and electrochemical technologies.

## Abstract 2

An optimization tool is presented by coupling a genetic algorithm with a pore network modeling framework for the bottom-up design of porous electrodes for a broad range of operating conditions, electrolyte chemistries, and reactor designs.

## Graphical abstract

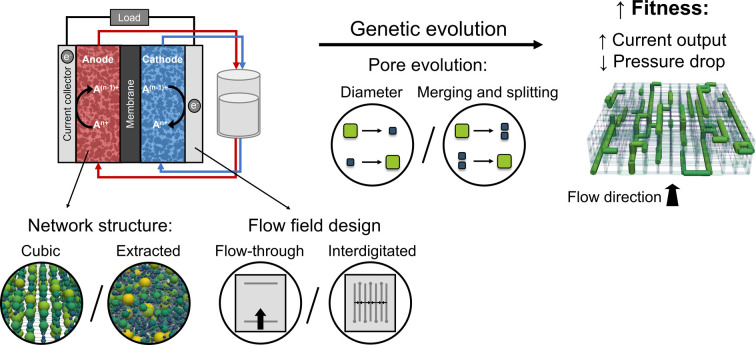

## 1. Introduction

Porous electrodes are integral reactor components in redox flow batteries (RFBs) and are essential to the battery performance, durability, and costs.^1,2^ The porous electrode provides the active surfaces for the electrochemical reactions, controls the distribution of the liquid electrolyte throughout the reaction zones, and conducts electrons and heat.^3–6^ Off-the-shelf porous electrodes are carbon fiber-based mats that are generally repurposed from more mature electrochemical technologies such as low-temperature fuel cells and have not been tailored to sustain liquid-phase electrochemistry.^1,7^ Thus, for RFBs to become a cost-competitive energy storage technology, porous electrodes tailored to specific flow battery chemistries and flow reactors must be designed and manufactured.^1,8,9^ However, because of the convection-enhanced nature of RFBs, the porous electrode design becomes challenging as contradictory requirements must be satisfied, including low pumping power, high electrochemical surface area, and facile mass transport.^2,5,10^ Hence, to solve the complex design requirements, advanced optimization strategies could be applied to design porous electrodes from the bottom-up.^11–13^

Genetic algorithms (GAs) are promising for exploring a broad geometrical design space for the optimization of porous electrodes. GAs are probabilistic global optimization techniques inspired by the theory of evolution that enable heuristic optimization of a given design space based on a fitness function.^14–17^ Therefore, GAs require only one objective function, can be parallelized, and have a large solution space. These unique features have motivated the application of GAs to a wide variety of research fields, including the integration of electrochemical numerical frameworks for the optimization of RFB parameters and conditions^18,19^ and electrode structures.^11–13^ For the optimization of the electrode geometry, a GA should be coupled to pore-scale simulations to capture the relationship between the battery performance and electrode microstructure in a computationally light manner. To this end, pore network models (PNMs) can be used as they capture microstructural effects at the mesoscale whilst being computationally light, with a limited loss in computational accuracy,^20^ owing to geometrical simplifications of the pore network.^8,21–23^ In PNMs, the pore space is captured by a combination of pores and throats, allowing the porous structure to be represented as a set of finite discrete points, enabling the mapping of the pores into a population in the GA. The coupling of PNMs and GAs has been used in other research fields including the extraction of pore networks of porous rock samples for petroleum recovery.^11,24–26^ However, the coupling of GAs with PNMs for the bottom-up design and optimization of electrode microstructures for RFBs, without the requirement of large datasets for optimization, remains largely unexplored.

**Fig. 1:**
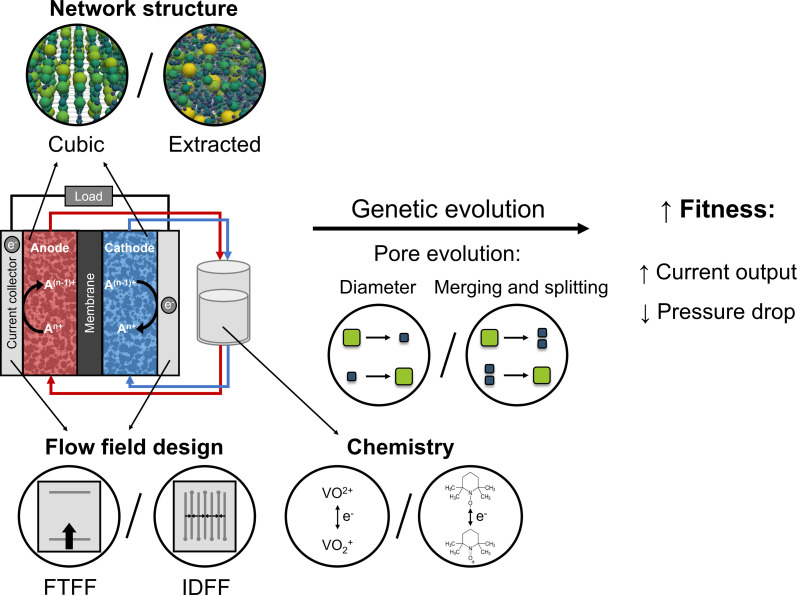
Schematic overview of the outline of this work, including a single-electrolyte flow cell, the starting networks used in the optimization (artificially generated cubic network and the X-ray tomography extracted paper electrode), the flow field designs (flow-through and interdigitated) used in this study, different chemistries (sluggish vanadium- and facile nitroxyl-based electrolytes), and pore evolution approaches (based on a pore diameter evolution and pore merging and splitting), to obtain an electrode with increased fitness by an enhanced current output and/or lower pressure drop.

The concept of combining a genetic algorithm with PNMs to optimize three-dimensional microstructures for flow battery electrodes was demonstrated in our previous work for cubic networks with fixed pore positions.^13^ The performance of the individual networks was evaluated by a fitness function that maximizes the electrochemical power and minimizes the pumping power of the networks. Here, we extend the optimization framework to include more design flexibility by extending beyond fixed pore positions through the integration of merging and splitting of pores (Fig. 1). In addition, we evaluate the optimization algorithm for structures with diverse morphologies such as X-ray tomography-extracted networks of commercial electrodes translated into pore networks, which have been proven to represent the microstructural properties of the electrodes reasonably well.^21^ By optimizing a commercial electrode, we show that we can obtain high-performing electrodes with improved electrochemical performance and lower pumping losses than benchmark materials. Furthermore, we investigate the effect of operation conditions by evaluating two redox chemistries, VO^2+^/VO_2_^+^ and TEMPO/TEMPO^+^, and two prevailing flow field geometries, a flow-through flow field (FTFF) and an interdigitated flow field (IDFF).

In this work, we first describe the modeling framework including the network generation, the coupling of the GA with the electrochemical algorithm, the genetic operations included in the GA, and the operating parameters investigated. Second, we show the geometrical evolution for the addition of pore merging and splitting as an additional mutation operation. Third, we deliberate on the influence of the network structure of the initial population on the fitness evolution and assess the impact of the flow field geometry on the structure evolution. Fourth and last, we perform the electrode optimization for two redox chemistries to investigate the importance of the starting network and the specific reactor architectures and operating conditions. This study, although applied to redox flow batteries here, shows the potential of optimization by genetic algorithms to design and optimize porous materials for a wide variety of convection-enhanced electrochemical applications. Furthermore, this work emphasizes the importance of co-designing electrodes and flow fields and assesses the sensitivity of genetic algorithms to optimization definitions.

## 2. Model development

Optimization by GAs can be applied to evolve a population of candidate solutions to increasingly better sets of solutions for the given design space. The principle is based on natural diversity and selection by the recombination of good building blocks. The concept of combining a GA with PNMs is described in our previous work for cubic networks with fixed pore positions,^13^ and extended upon in the present study. The pumping and electrochemical power defining the selection criteria were solved using a developed and validated PNM,^21^ that uses the open-source framework OpenPNM.^23^ A PNM was employed because of the low computational cost required to simulate local transport within porous electrodes, where the void space is approximated by spherical pores and cylindrical throats. The bulk solution is assumed well-mixed in each pore and the transport is dictated by the throats in the pore network. The idealization of the void space allows a simplification of the model equations while retaining microstructural information,^23^ thereby capturing the electrochemical performance in realistic electrode structures.^21,22,27,28^ The PNM was developed for single-electrolyte flow cell designs in discharge mode with the co-flow operation of the anodic and cathodic half-cells and thus optimizes the electrode in only one half-cell, assuming perfect electrode wetting. The required computational time for the reference system on a single Intel® Core™ i7-8700K CPU was ∼48 hours for 1000 generations based on 50 individuals and 10 parent networks (∼2 seconds per network), which can be significantly reduced when using multiple computing cores. After parallelizing the fitness function evaluation and running it on a cluster with 50 cores, the required computational time was reduced to ∼27 hours for the reference system.

**Fig. 2:**
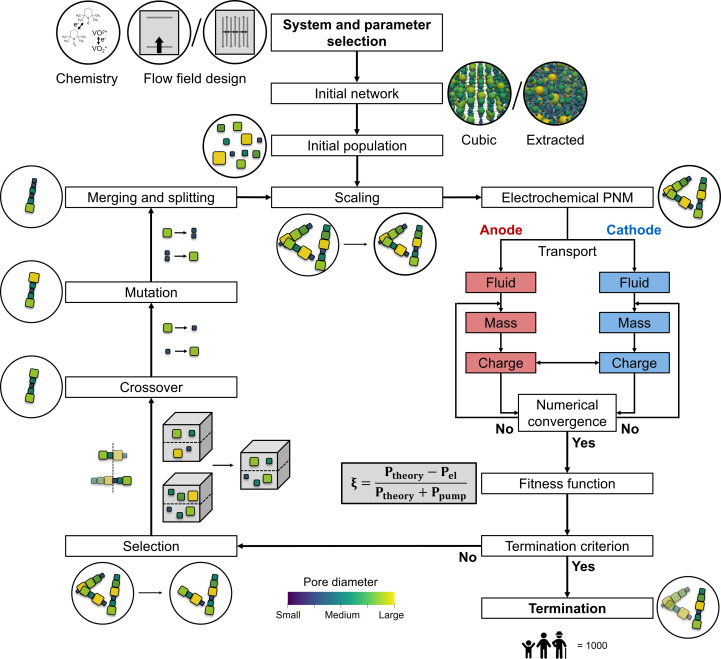
Schematic flowchart of the genetic algorithm used in this work, including a simplified flowchart of the integrated pore network model, together with illustrations of the chemistries, flow fields, initial networks, network structures after each operation, and the merging and splitting, crossover, and mutation operations.

The presented coupled optimization routine consists of nine steps: the network generation, initialization, volume scaling, the electrochemical PNM, parent selection, crossover, mutation, merging and splitting, and termination (see Fig. 2 for the schematic overview of the GA with the integrated PNM). In the network generation stage, the type of network was selected (cubic or extracted) of which a random set was generated in the initialization step defining the first population. In the first step of the iterative GA-PNM, volume scaling was performed to ensure a meaningful comparison at constant electrode porosity between the different individuals in a population and was repeated for each generation. Thereafter, the networks were evaluated based on the electrochemical PNM and a fitness function. Successively, the fittest individuals (*i.e.*, parents) in the population were selected and defined as the next population by inheriting the information of two parent networks with a crossover step. Afterward, stochastic changes were made in the networks of the new population by pore mutation, after which the pores in the network had a chance to merge and split, resulting in the next generation. The iterative GA-PNM was repeated until the termination criterion was achieved based on the total number of generations. The population size, number of parents, mutation range and probability, and merging and splitting probability and ratio were initially chosen arbitrarily or inspired by the work of Grefenstette.^29^ The number of generations and network size strongly impact the required computational time, which was set to only 1000 generations and a small network size (∼500 × 500 × 200 μm^3^) to showcase the principle of the GA-PNM without being computationally intensive and requiring high computing resources. Finally, the parameters defining the evaluation criteria of the PNM were based on our previous works.^13,21^ All reference parameters used in this work for the GA can be found in Table 1.

**Table 1:** Reference parameters used for the optimization study

Parameter	Value
Number of generations	1000
Number of offspring	50
Number of parents	10
Mutation range	0.1
Mutation probability	0.05
Merging and splitting probability	0.1
Merging and splitting ratio	0.5

### 2.1. Network generation

Cubic ordered lattices can be used as a first step to assess the impact of the GA on the electrode performance and to help evaluate the influence of optimization parameters on the genetic evolution. To induce randomization and complexity in the artificial networks, networks created by Voronoi tessellation of arbitrary base points could be used as starting geometries. Nevertheless, these simplified structures do not capture the permeability and electrochemical performance of fiber-based porous electrodes. To mimic the complex pore and throat locations, connections, and hydrodynamic properties of real porous electrodes, networks extracted from tomographic imaging should be investigated.

After the generation of the network structures with diverse morphologies, geometrical properties were attributed to the pores and throats in the network with geometry objects. The geometry objects are a subclass within OpenPNM that can be assigned to parts of the modeling domain. In this work, the geometric StickAndBall approach was applied to all networks, which handles pores as spheres and throats as cylinders in the generated networks for which geometrical properties can be calculated. A detailed description of the artificial and extracted network generation can be found in Section A1 in the Appendix.†

The geometrical property worth mentioning here is the pore internal surface area (*A*_p_), described by the OpenPNM geometry functions, defined by subtracting the throat cross-sectional area (*S*_T_) of *N*_T_ number of neighboring throats from the pore surface area obtained with the pore diameter *d*_p_ (eqn (1)). This definition, used for cubic networks unless stated otherwise, is a simplification of the pore internal surface area as the curvature of the intersection between the pore and throat was not considered. To this end, this definition cannot be applied to the extracted electrode as a negative internal surface area is obtained (connectivity >6 resulting in a large throat cross-sectional area subtraction, Section A4.1†) resulting in the optimization of small pores. Hence, eqn (2) was used for the extracted electrodes, which results in a underestimation of the internal surface area in comparison with literature values (*i.e.*, 2.2 × 10^4^ m^−1^ using eqn (2)*vs.* 7.2 × 10^4^ m^−1^ obtained in the literature for the Freudenberg H23 electrode^6,21^). As the surface area of the extracted electrode is in the same order of magnitude as measured experimentally, we believe that for this computational work eqn (2) is a reasonable approximation of the surface area for the extracted electrode. However, we must point out that the simplified geometrical properties are a limitation of the PNM when capturing the exact properties, such as the electrochemically active surface area, compared to practical systems. Nevertheless, the distinct electrode structures should mainly be compared in terms of their optimization trends rather than quantitatively. The reader is referred to Section A4.1† for a detailed discussion of the surface area definition.1
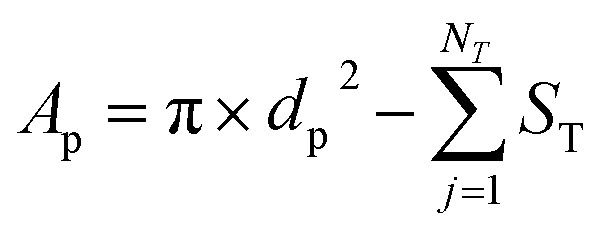

2 *A*_p_ = π × *d*_p_^2^

The geometrical definitions used strongly affect the optimization as they directly impact the electrochemical and pumping power. Therefore, in Section A4,† the sensitivity of the optimization depending on the definitions of the internal surface area, throat diameter, and electrode size were analyzed. Changing the definitions affected the electrode optimization, showing the importance of selecting the appropriate geometrical definitions for the optimization. However, all studies did result in optimized structures with a bimodal pore size distribution with longitudinal transport pathways in the flow direction that reduced the pumping power. As simplified descriptions can strongly over/underestimate the performance as well as alter the optimization, geometry definitions need to be identified that consider the manufacturing method of the final electrodes. For example, when optimizing structures that will be 3D printed, the surface area definition could consider all pore and throat walls as internal surface area, but this is beyond the scope of this work.

The network properties of the cubic and extracted structures are given in Table 2, where, in this work, small three-dimensional electrode structures were optimized with electrode geometrical areas of approximately 500 × 500 μm^2^. To allow comparison between the two network structures, the network shape and spacing of the cubic structure were based on the microstructural properties of the off-the-shelf Freudenberg H23 paper electrode (Fuel Cell Store, 80% porosity) with a median pore size of 20 μm and a measured thickness of 210 μm.^6,10,13,21^

**Table 2:** Network properties used for the optimization study for the cubic and extracted network for the FTFF. The networks were twice as wide for the IDFF

Parameter	Cubic	Extracted	Unit
Porosity	54	51	%
Network shape	[13, 13, 4]	—	—
Number of pores	676	3348	—
Number of throats	1755	10 171	—
Network size	580 × 580 × 220	500 × 500 × 198	μm^3^

### 2.2. Initialization

The initial population was created based on the network properties (Table 2) and the number of offspring networks (Table 1). For the cubic network, the diversity of the offspring comes from the randomization created by the pore seed, whereas for the extracted networks it is introduced by a mutation of the original extracted network, depending on a mutation factor and range, see Section 2.7.

### 2.3. Network scaling

To enable meaningful comparison between individuals in each generation and between generations, a network scaling step was performed where the pore diameters were uniformly scaled to a reference network. The network scaling was based on the total pore and throat volume, maintaining a constant network porosity during the evolution. The reference networks used for this scaling step depended on the network type. For cubic networks, the reference network was based on the pore-to-throat ratio described by Sadeghi *et al.* where they translated a commercial porous electrode to a pore network consisting of pores with a diameter of 15.6 μm and throats with a diameter of 20 μm.^22^ For the extracted networks we used the initial extracted structure, scaled with the definitions described in Section A1.†

### 2.4. Electrochemical algorithm

The electrochemical algorithm developed and validated in our previous work^21^ was integrated into the GA (Fig. 2) to evaluate the performance of the individuals in every generation, with the electrochemical power and pumping power as outputs. The PNM was designed for single-electrolyte flow configurations with a constant state-of-charge of 50% and an open circuit voltage of 0 V to study electrode overpotentials in isolation without secondary effects including membrane crossover and changing state-of-charge.^1,5^ The model solved the local fluid transport and the coupled mass and charge transport within both half-cells using an iterative approach, where the locations of the flow field channels, ribs, current collectors, and membrane were defined by boundary conditions, see Fig. A2.† In this work, two flow field configurations were studied, an FTFF and an IDFF. The FTFF was previously validated for two electrode structures and two electrolytes and showed a near-unidirectional velocity distribution from the inlet to the outlet channel over the entire electrode length.^21^ The IDFF on the other hand was validated in our other work and features a unique velocity profile from the inlet to the outlet channel through the electrode over a rib.^30^ More information regarding the iterative PNM can be found in Section A2† and in our previous works.^13,21,30^

### 2.5. Parent selection

The electrochemical performance and pressure drop of the individual network structures were analyzed in the GA-PNM with a fitness function (*ξ*) to select the best-performing networks for reproduction. The fitness function considers the maximum thermodynamic electrochemical power (*P*_max_), electrochemical power loss (*P*_el_), and pumping power (*P*_pump_), where the maximum fitness is achieved when the pumping losses are zero. In this work, the electrochemical losses were evaluated at a fixed overpotential (*E*_losses_) of 0.5 V, corresponding to 40% of heat loss during cell discharge, and define the combined overpotential (activation, ohmic, and mass transfer) present in the single-electrolyte cell. By fixing the overpotential, we aim to increase the maximum electrochemical power by achieving a higher total current (*I*). Moreover, the open circuit cell potential (*E*_cell_) was considered 1.26 V for the reference system,^31^ resulting in a maximum achievable fitness value of 0.603. Further, as we optimized electrode structures for single-electrolyte cell designs, we assumed that the overpotentials resulting from the other redox pair were identical in this theoretical exercise. The pumping power required to pump the liquid electrolyte through the pore network depends on the electrolyte flow rate (*Q*), which was set at 1.5 cm s^−1^, the pressure drop (Δ*P*), and a pumping efficiency (*η*_p_), set to 0.9.^32^ Hence, the fitness function was defined by eqn (3) by dividing the electrochemically generated power by the summation of the maximum electrochemically generated power and pumping requirements,^13,32,33^3
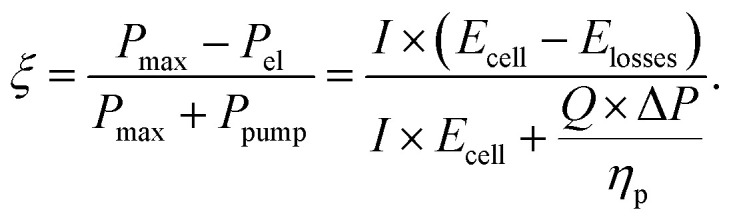


The individuals with the highest fitness value (*ξ* → 0.603) were selected as parent networks and were subjected to crossover in the next step. Finally, the total percentual fitness increase over the generations can be calculated according to (*ξ*_n_−*ξ*_1_)/*ξ*_1_ × 100%, with *ξ*_n_ the fitness of the best-performing network in the last evaluated generation, and *ξ*_1_ the fitness of the best network of the first generation.

Because the objective of the GA is to optimize electrode structures based on a defined fitness function, a suitable definition needs to be determined that considers the objective of the optimization. In Section A4.4† the effect of the fitness function on the optimization was investigated by performing optimization studies with only the pumping power or only the electrochemical power considered. From this study, we found that when only optimizing for the electrochemical power, the pumping power remained unoptimized. On the contrary, when optimized for the pumping power alone, the optimization was comparable to that with eqn (3), suggesting that this fitness definition steers towards the optimization of the pumping power over the electrochemical power. Furthermore, future work could investigate alternative optimization parameters, such as the overpotential and flow rate, or the pumping-corrected voltage efficiency and areal power density, instead of the current density and the pumping power. To change the objective of the optimization, only the electrochemical PNM must be adapted to solve for the desired parameters, which thereafter should be incorporated in the fitness function definition. Finally, the form of the fitness function could be reconsidered, for example by defining the fitness function based on the difference in performance between the new and initial generation.

### 2.6. Crossover

The crossover operator was responsible for the recombination of the parent networks into new offspring networks, defining the next generation. These new offspring networks were stochastically generated as the parent networks were arbitrarily selected from the mating pool and the crossover point or coordinate was randomly selected. The crossover was performed by two different methods in this work: by single-point or coordinate-based crossover. The single-point crossover was used for networks with fixed pore coordinates by selecting a random pore between the first and final locus of the pore diameter array, defining the crossover point.^34^ Thereafter, the new offspring networks were created by inheriting the geometrical information, including pore and throat diameters, of the first parent between the first pore and the crossover point, and that of the second parent between the crossover point and final pore. Alternatively, the coordinate-based crossover was used to handle pore networks with a varying number of pores and pore coordinates and can thus be applied to networks that underwent pore merging and splitting. In this crossover approach, a plane at half the electrode width was selected as the crossover plane, which splits the left and right parts of two parent networks. Then, the left part of one parent and the right part of another parent were stitched together to form the new offspring networks. The old throats at the plane boundary were deleted and new throats were reestablished between the two parent networks at the plane boundary, where the nearest pores were connected and the number of original connections remained constant.

### 2.7. Mutation

The mutation operator is a fundamental instrument to ensure population diversity. In this work, the mutation was based on randomly altering the pore diameters of the offspring networks. To control the mutation, a mutation probability and range were introduced. The mutation probability defined the probability of mutation for each pore and the mutation range (*σ*_M_) controlled the severity of the mutation. When a pore was selected for mutation, a random mutation value (*c*_M_) was stochastically assigned to the pore within the boundaries defined by the mutation range: (1 − *σ*_M_) ≤ *c*_M_ ≤ (1 + *σ*_M_). Thereafter, the pore diameter was mutated to a new value (*d*^M^_p_) by multiplying the mutation value with the old pore diameter (*d*^o^_p_):

4 *d*^M^_p_ = *c*_M_ × *d*^o^_p_.

### 2.8. Merging and splitting

In our previous work,^13^ the pore locations and the network connectivity were fixed, restricting the networks from evolving into more geometrically detailed structures. Therefore, we incorporated merging and splitting of pores in the GA to broaden the design space for evolution by allowing pore mobility. The merging and splitting of pores were defined by a merging and splitting probability and ratio. When the ratio is 0.5, there is an equal chance for pore merging as for pore splitting, and the number of pores and throats is kept nearly constant over the evolution.

Pore merging was performed based on an built-in OpenPNM function in which two or more pores can be combined at the center of the selected pores. In this GA, only two neighboring pores were allowed to merge, where the neighbor of the selected pore with the smallest pore diameter was chosen for merging. Furthermore, the pore volume was defined as the summation of the two pores, ensuring a constant total pore volume. Thereafter, the throat connections were reestablished between the neighbors of the merged pores and the new pore, decreasing the number of throats by one.

With pore splitting, on the other hand, the selected pore was split into two pores with equal pore volume. The new pore locations were stochastically determined within half a maximum pore diameter distance in each direction and a new throat was formed between the two new pores. The old throat connections with the neighboring pores of the split pore were reestablished to the closest of the two new pores, increasing the total number of throats in the network by one. Finally, it must be noted that for both pore merging and splitting, the new pore locations must be checked for pore overlap with nearby pores and if the new pores are within the specified network dimensions. In case of pore overlap, the pore locations were updated by stochastically altering their coordinates.

### 2.9. Termination

The iterative GA-PNM was repeated according to the scheme presented in Fig. 2 and was only terminated when the maximum number of generations was achieved. We elected this termination criterion to enable a meaningful comparison^13^ between networks generated at different operating conditions, evolutionary parameters, electrolyte properties, flow field architectures, and network geometries.

### 2.10. Operating parameters

In this study, two distinct redox chemistries were analyzed: the vanadium chemistry based on the vanadyl and pervanadyl ions (VO^2+^/VO_2_^+^) and an organic electrolyte with the 2,2,6,6-tetramethylpiperidine-1-yl)oxyl radical and 2,2,6,6-tetramethyl-1-piperidinyloxy-oxo ion (TEMPO/TEMPO^+^). The electrolyte and electro-kinetic properties of the distinct redox couples for a single-electrolyte cell design are given in Table 3, obtained in our previous works.^13,21^ The open circuit cell potentials corresponding to each redox pair were based on full battery systems: the all-vanadium,^31^ or the 4-hydroxy-2,2,6,6-tetramethylpiperidine-1-oxyl (TEMPO-OH) and methyl viologen system.^35^ To comply with the dilute electrolyte assumption used in the PNM (*i.e.*, migration was not considered^36^), we selected relatively low inlet concentrations of 100 mol m^−3^ per species in an excess supporting electrolyte solution of 1000 mol m^−3^ for the investigated electrolytes. We elected the vanadium electrolyte as our reference system which was used in the optimization unless stated otherwise. This system was selected as it is state-of-the-art^37^ and features a kinetically sluggish redox couple,^5,38^ which allowed the optimization of both the electrochemical performance (*i.e.*, available surface area and mass transfer) and parasitic pumping losses.^39^ Even though single-electrolyte flow cell designs were used in this study, the overarching trends obtained can guide the design of next-generation porous electrode designs, depending on the system configuration (flow field design and operating conditions) and electrolyte properties.

**Table 3:** Electrolyte and electro-kinetic properties for the VO^2+^/VO_2_^+^ and TEMPO/TEMPO^+^ chemistries in both half-cells based on the data from,^13,21^ where 1 and 2 refer to the distinct species

Parameter	Vanadium	TEMPO	Unit
Density of the electrolyte, *ρ*	992	852	kg m^−3^
Viscosity of the electrolyte, *μ*	8.9 × 10^−4^	3.4 × 10^−4^	Pa s
Diffusion coefficient, *D*_1_	2.11 × 10^−10^	1.3 × 10^−9^	m^2^ s^−1^
Diffusion coefficient, *D*_2_	2.11 × 10^−10^	1.3 × 10^−9^	m^2^ s^−1^
Inlet concentration, *c*_1,in_	100	100	mol m^−3^
Inlet concentration, *c*_2,in_	100	100	mol m^−3^
Supporting electrolyte concentration	1.0 M H_2_SO_4_	1.0 M TBAPF_6_	
Bulk electrolyte conductivity, *σ*	28	1.99	S m^−1^
Cathodic transfer coefficient, *α*_c_	0.42	0.5	—
Anodic transfer coefficient, *α*_a_	0.42	0.5	—
Exchange current density, *j*_0_	0.39	460	A m^−2^
Electrolyte velocity, *u*	1.5	1.5	cm s^−1^
Cell potential, *E*_cell_	1.26	1.25	V
Overpotential, *E*_losses_	0.5	0.5	V

## 3. Results and discussion

### 3.1. Beyond fixed lattice positions

In our previous work,^13^ the pore positions were fixed on a cubic lattice with a connectivity of 6, restricting the evolutionary freedom of the optimization. To this end, we included pore merging and splitting, allowing for a change in the number of pores and their location. This additional network mutation step results in more evolutionary freedom but comes at the cost of an increased optimization complexity. As there is another randomization step involved, the algorithm requires more generations to achieve the same fitness increase but allows for more realistic network structures (*i.e.*, closer to commercial fibrous electrodes employed in RFBs). The results of the optimization with merging and splitting are shown in Section A5.† We compare the performance of the GA-PNM with only mutation, only merging and splitting, and a combination, showing the impact of both mutation operators on the optimization of the networks. Moreover, the GA-PNM was run without any mutation operator (no mutation and no merging and splitting, *i.e.*, only crossover), which resulted in minimal structure optimization, showing that a mutation operator is key for structure optimization.

**Fig. 3:**
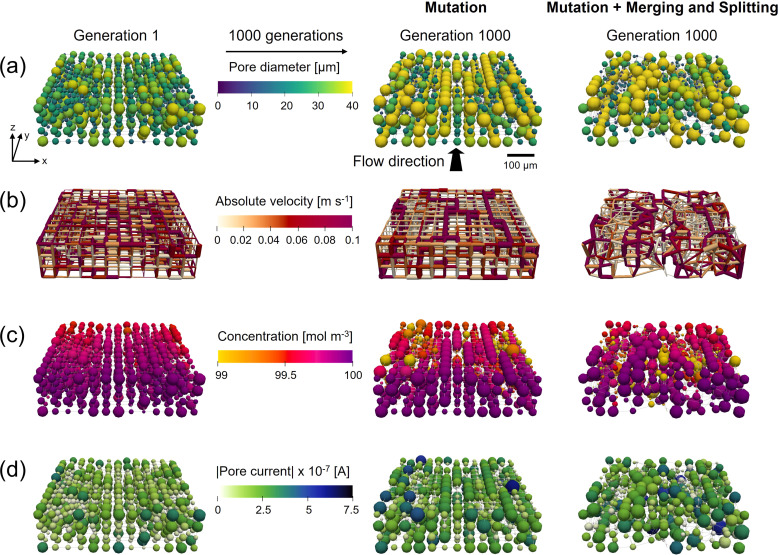
The geometrical evolution of cubic networks with only a mutation operator, and a combination of a mutation and a merging and splitting operator. The networks of the first and final (1000th) generations are shown, displaying the: (a) pore diameter evolution, (b) the throat absolute velocity, (c) the reactant pore concentration, and (d) the absolute pore current. With the flow in the *y*-direction and the thickness in the *z*-direction with the membrane facing to the top.

The results in Section A5.1† show that the mutation step results in the main fitness improvement by allowing the formation of the electrolyte transport pathways in the flow direction consisting of large pores (36–40 μm), connected by large throats (29–32 μm, Fig. 3a), enhancing both the electrical and pumping power. Merging and splitting alone, on the other hand, results in a fitness improvement (21%), but the pores do not form well-defined transport pathways because of the randomized locations of merging and splitting and due to the absence of mutation. When combined, transport pathways^13^ are formed (visualized in Fig. A4 in Section A3†), decreasing the pumping power and increasing the electrical performance. However, the total fitness improvement is lower (31% *vs.* 42%) compared to the case with only mutation. To this end, the effect of the merging and splitting ratio is investigated in Section A5.2† where we found that the ratio between merging and splitting is an important parameter that can steer the electrode optimization toward the formation of networks with more or fewer pores than the starting network.

The network properties of the cases with mutation and combined with merging and splitting are compared in Fig. 3. In Fig. 3a the pore diameter evolution is visualized, presenting the randomization of the merging and splitting operator and the formation of the longitudinal transport pathways for both cases.^13^ These transport paths consist of interconnected large pores, which, by geometrical definition, have throats with a large diameter connecting the pores. The large throats feature a high absolute velocity (Fig. 3b) and are driving the transport of the electrolyte through the electrode, decreasing the pressure drop. Alongside the electrolyte transport pathways, isolated large pores are present with a large surface area (eqn (1)) that allow more redox reactions to take place, decreasing the concentration locally (Fig. 3c) and resulting in a high current output (Fig. 3d). The outlet concentration of the reactant in Fig. 3c is high because of the small network sizes (∼500 μm). If we run a network-in-series approach on the networks to simulate a larger electrode size (>10 cm^2^), the species outlet concentration is much lower as discussed in our previous work.^21^ When comparing Fig. 3 for the two cases, the main difference is in the randomization of the structures as the overall optimization trends remain comparable, such as the formation of transport pathways and higher reaction rates near the membrane interface. To conclude from this comparison, we find that mutation is necessary to speed up the optimization of the networks, whereas merging and splitting adds an additional mode of randomization, allowing for more realistic networks to be formed, but at the cost of a slower fitness optimization.

### 3.2. Impact of the flow field design

Besides the addition of merging and splitting to allow for more realistic networks, we investigate the approach of starting the optimization from morphologically distinct electrode structures. To this end, we study the commercial Freudenberg H23 carbon paper, extracted using X-ray computed tomography and translated to a pore network. In our previous work, we have shown that extracted pore networks represent the microstructural properties of the electrodes reasonably well.^21^ By optimizing benchmark materials we aim to demonstrate that optimization algorithms can enhance realistic electrode structures beyond carbon fibrous electrodes to provide new insights on high-performing electrodes.

**Fig. 4:**
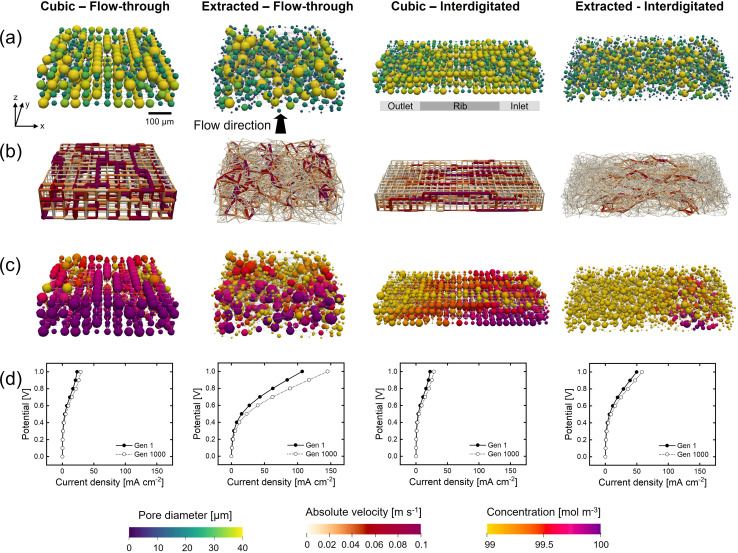
The geometrical evolution of cubic and extracted networks for the flow-through and interdigitated flow fields with a mutation operator and the vanadium electrolyte. The networks of the last (1000th or 200th) generation are shown, displaying the: (a) pore diameter, (b) the throat absolute velocity, (c) the reactant pore concentration, and (d) the polarization curve of the first and last generation. With the flow in the *y*-direction and the thickness in the *z*-direction with the membrane facing to the top.

The initial network structure strongly impacts the structure optimization in terms of the starting performance (fitness value and electrochemical and pumping power) and structure optimization flexibility (number of pores, pore locations, and maximum pore diameter), especially when bound to fixed lattice positions. Therefore, we compare the networks without merging and splitting to identify how distinct fixed lattice positions impact the electrode optimization. Furthermore, to show the system dependency of the optimization framework and thus the need to engineer electrodes depending on their application requirements, we incorporated another flow field design into the framework, *i.e.*, the interdigitated design. In our previous works,^30,40^ as well as in other literature,^41,42^ the interplay between the electrode and flow field was proven to be imperative to the RFB performance. For example, Muñoz-Perales *et al.* found that for Fe^2+^/Fe^3+^ single-electrolyte flow cells in combination with an FTFF, woven bimodal electrodes are favorable. Whereas when using IDFFs, unimodal paper electrodes result in enhanced flow cell performance.^41^ Hence, in this work, we elect to investigate the GA-PNM structure optimization with the FTFF and IDFF in combination with cubic and extracted networks. The optimized structures are shown in Fig. 4 and the structure evolution and optimization values are presented in Sections A6 and A7.†

For the FTFF, both the cubic and extracted networks show an improvement in fitness over the generations, starting from different fitness values, where the fitness evolution is unique for each optimization case with diminishing returns after 100 and 400 generations, respectively. Moreover, both networks evolve towards a bimodal structure with large interconnected pores in addition to small pores (Fig. 4a and A21†). The bimodal structures consist of large connected pores in the flow direction, linked by throats of a large diameter and a high absolute velocity (Fig. 4b), responsible for the electrolyte transport and thus the decrease in pumping power. The decrease in pumping power is the most prominent for the extracted network (65% compared to 54% for the cubic network) related to the higher absolute pumping power required because of the less ordered throats (in-plane) compared to the cubic network. Moreover, the extracted network has the highest increase in electrical power upon comparison with the same surface area definition (22% *vs.* 3.6% for the cubic network).

Furthermore, with the FTFF the extracted network shows a higher species conversion in the smaller pore segments (Fig. 4c) compared to the cubic network. Especially for electrolytes with sluggish kinetics but high ionic conductivity, the large number of small pores in the extracted network is beneficial. In the electrochemical PNM used, the mass transfer coefficient is a function of the diffusion coefficient and the pore radius and is thus velocity-independent. Hence, smaller pores have a higher mass transfer coefficient and thus a higher species conversion per unit volume (Fig. 4c). Even though small pores have a lower surface area, the extracted network has the same total surface area compared to the cubic network (1.4 × 10^−6^ m^2^*vs.* 1.5 × 10^−6^ m^2^) because of the large number of small pores, resulting in enhanced mass transfer in the extracted network. The high number of small pores results in a significantly higher limiting current density, as seen in Fig. 4d. Moreover, as a result of the structure optimization by the formation of a bimodal pore size distribution with small (2–20 μm) and large pores (40–60 μm) and large throats (20–40 μm), there is a strong reduction in the activation (7% at ∼60 mA cm^−2^, due to the increase in internal surface area of 31%), concentration (24% at ∼60 mA cm^−2^), and ohmic overpotentials (41% at ∼60 mA cm^−2^). For the cubic network, the increase in performance is caused by the reduction in the activation and concentration overpotentials (at ∼20 mA cm^−2^ a 4% reduction in activation overpotential and a 41% reduction in concentration overpotential) as a result of the increase in internal surface area (34%), yet the ohmic overpotential is not significantly reduced. The optimization of the ohmic overpotential in the extracted network is expected to come from an increased ionic flux towards the membrane due to the formation of large throat segments (20–40 μm) with higher electrolyte velocity in the through-plane direction (Fig. 4b). The larger throats result in a greater penetration of the reaction front into the electrode for reactions, corroborated by the high hydraulic conductance of these larger throats through the network in the last generation^21^ (Section A6†). Combined with the high conductivity of the electrolyte, this provides the optimized extracted network with the largest current output at 1 V. Thus, it is anticipated that due to a large number of pores and their random orientation, the extracted network has a greater optimization chance for both the electrical power and pumping power compared to the cubic network with fixed lattice positions.

The fitness, electrical, and pumping power evolutions with the IDFF portray similar trends compared to those with the FTFF, as well as the percentual increases over the generations. When comparing the networks, the electrical power and surface area of the networks are about 2x greater with the IDFF because of the twice as wide network size. Moreover, noteworthy dissimilarities can be observed in the absolute pumping power required. For both the cubic and extracted networks, the pumping power increases when utilizing IDFFs because of the twice-as-wide electrode. The required pumping power for a larger electrode (>10 cm^2^) however would result in a much higher pressure drop for the FTFF because of the longer electrolyte pathway through the electrode compared to that for the IDFF. Where the cubic structure has a lower pumping power compared to the extracted network combined with an FTFF (5.2 μW *vs.* 7.8 μW), the extracted structure shows a reduced pumping power after optimization with the interdigitated design (9.0 μW *vs.* 6.0 μW). In our previous works, we observed that the pressure drop through carbon paper electrodes is strongly reduced when using an IDFF,^30^ whereas the pressure drop was even increased through 3D-printed model grid electrodes compared to FTFFs.^40^ The findings in this work are in line with our previous works as the optimized cubic structure with in-plane high-velocity pathways is favorable with FTFFs, whereas the (optimized) paper electrode results in a lower pressure drop combined with IDFFs because of the combined in-plane and through-plane fluid flow over the rib.

Furthermore, because of the distinct flow distributions of the flow fields, the GA-PNM optimizes the electrodes to significantly different structures (Fig. 4a). For both the cubic and extracted networks, large pores connected by throats with a large diameter are formed in the flow direction (*i.e.*, from the inlet to the outlet channel over a rib). These electrolyte transport pathways result in throats with a high velocity from the inlet to the outlet (Fig. 4b), decreasing the pressure drop. In addition to the electrolyte transport pathways, with the IDFF stagnant zones are formed near the membrane under the inlet and outlet channels with low electrolyte velocities (Fig. 4b) and high species conversion (Fig. 4c).^30^ It must be noted that the species conversion is higher for the IDFF compared to the FTFF because of the electrode width and electrolyte pathway and because the results are shown without the network-in-series approach for the FTFF.^21^ Moreover, as shown in the polarization curves (Fig. 4d), the ohmic overpotential is again reduced for the extracted network as the ionic conductance in the flow direction is improved for the optimized structure because of the formation of large throats (Section A6†), enhancing the ionic flux towards the membrane. When comparing the polarization curves in Fig. 4d, we find that the cubic structure shows similar performance in terms of the current output with both flow fields (29 mA cm^−2^ at 1 V), whereas the extracted structure shows a 2.5 times lower performance with the interdigitated design (∼58 mA cm^−2^*vs.* ∼145 mA cm^−2^ at 1 V). The formation of the stagnant zones under the inlet and outlet channels with high species conversion, for both the initial and final networks, is anticipated to cause increased activation overpotential with the IDFF compared to the FTFF. These regions occupy a significant part of the internal surface area but feature a low electrolyte velocity (Fig. 4b) which could lead to inferior electrolyte replenishment.

In conclusion, when comparing the electrode optimization for FTFFs and IDFFs, similar trends can be observed. The optimization with both flow fields results in structures with a bimodal pore size distribution with large pores connected by throats with a large diameter, responsible for the electrolyte transport and causing a reduction in the pumping requirements of 55–77% for all investigated systems. Moreover, the electrical power is improved by 22–39% caused by an increase in the internal surface area and improved ionic conductance. In practice, utilizing IDFFs is expected to reduce the pressure drop through larger electrodes (>10 cm^2^), especially for structures with small pores. On the other hand, the current output depends on the electrode length, as for FTFFs the species outlet concentration is strongly correlated to the electrode length, which is not the case for the IDFF. Therefore, when translating the optimization results to larger electrode sizes, it must be kept in mind that the tradeoff between the pumping power and electrochemical power will shift, as especially the pumping power will have a larger contribution when using an FTFF. We find that, when optimizing for only the pumping power (Section A4.4†), the optimization results in similar structures and we thus expect this electrode design also to be favorable for RFBs with larger electrode sizes. Finally, the distinct flow fields introduce unique electrolyte flow profiles through the electrode with an in-plane flow in the length direction for the FTFF and combined in- and through-plane flow in the width and thickness direction for the IDFF. Thus, the coupling of electrode design with specific flow field architectures is crucial and should be considered in future RFB electrode design.

### 3.3. Chemistry-dependent optimization

**Fig. 5:**
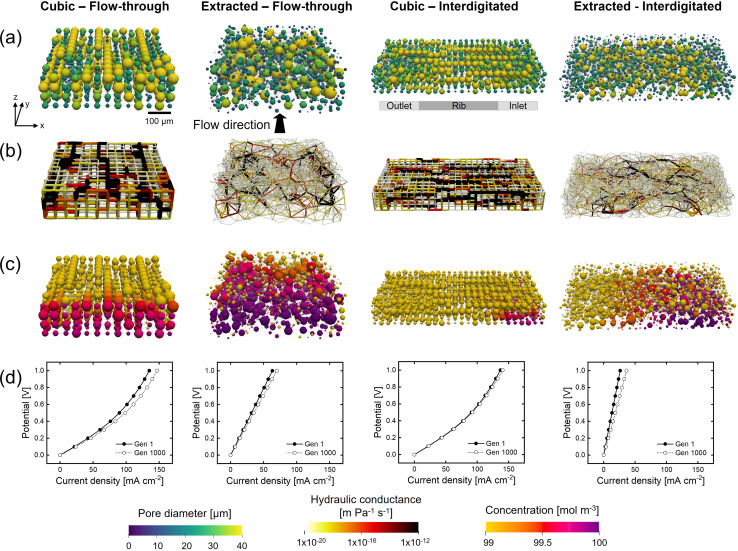
The geometrical evolution for the TEMPO-electrolyte for cubic and extracted networks and the flow-through and interdigitated flow fields with a mutation operator. The networks of the last (1000th) generation are shown, displaying the: (a) pore diameter, (b) the throat hydraulic conductance, (c) the reactant pore concentration, and (d) the polarization curve of the first and last generation. With the flow in the *y*-direction and the thickness in the *z*-direction with the membrane facing to the top.

The electrolyte chemistry dictates the electrochemical performance by, among others, the kinetic rate constant and ionic conductivity. The vanadium VO^2+^/VO_2_^+^ electrolyte features sluggish kinetics, with a low exchange current density of 0.39 A m^−2^, and a high ionic conductivity of 28 S m^−1^.^13^ Therefore, the GA prioritizes the electrode structure optimization for a high internal surface area for the vanadium electrolyte to decrease the activation overpotential over the enhancement of the through-plane ionic conductance to decrease the ohmic overpotential, especially for the cubic structure. Thus, we expect that the electrolyte chemistry can considerably influence the structure optimization and we therefore investigate the optimization of the non-aqueous TEMPO/TEMPO^+^ electrolyte^21^ with facile kinetics (exchange current density of 460 A m^−2^) and a low ionic conductivity (2.0 S m^−1^). The impact of the electrolyte on the structure evolution was assessed for both networks and flow fields in Fig. 5. The structure evolution and values can be found in Section A8.†

For both flow fields, the fitness of the networks with the TEMPO electrolyte is close to the theoretical maximum (*ξ* → 0.603) as both the pumping power and electrical power are improved. The pumping power is lower than for the vanadium electrolyte because of the lower electrolyte viscosity (3.4 × 10^−4^*vs.* 8.9 × 10^−4^ Pa s), yet the same percentual decrease in pumping power is obtained for both electrolytes (52–55% for the cubic structures with both flow fields and 65–66% with the FTFF, and 77% with the IDFF for the extracted networks). The electrical power is enhanced with the TEMPO electrolyte at 0.5 V applied potential (for the FTFF: 100 mA cm^−2^*vs.* 5 mA cm^−2^ for the cubic network and 35 mA cm^−2^*vs.* 23 mA cm^−2^ for the extracted network) because of the negligible activation overpotential due to the facile kinetics (Fig. 5d). For the FTFF to this end, the percentual increase in the internal surface area is lower for the TEMPO electrolyte (18% *vs.* 34% for the cubic and 25% *vs.* 31% for the extracted network), which translates to a lower percentual increase in the electrical power for the cubic (7.5% *vs.* 30%) and extracted networks (11 *vs.* 22%). Furthermore, facile kinetics give rise to a higher species conversion for the cubic networks as can be seen in the concentration profiles in Fig. 5c.

When evaluating the performance at 1 V applied potential in Fig. 4d and 5d for both flow fields, it is found that the cubic network has a considerably higher current output with the TEMPO electrolyte (140 mA cm^−2^) compared to the vanadium electrolyte (29 mA cm^−2^) because of the strong decrease in activation overpotential. Whereas the extracted network has a lower performance with the TEMPO electrolyte at 1 V (70 mA cm^−2^*vs.* 145 mA cm^−2^ for the vanadium electrolyte with the FTFF, and 36 mA cm^−2^*vs.* 58 mA cm^−2^ with the IDFF) due to an increased ohmic overpotential. These results support our claim on the importance of the operating conditions, reactor design, and electrolyte chemistry on the electrode selection and optimization, as the cubic network that performed significantly worse with the vanadium chemistry, outperforms the extracted network with the TEMPO chemistry. Even though the internal surface area is 1.6 x lower for the cubic network, the activation (0.04 V *vs.* 0.009 V at ∼70 mA cm^−2^ with the FTFF) and concentration overpotentials (0.04 *vs.* 0.006 V at ∼70 mA cm^−2^ with the FTFF) are larger, while the membrane resistance and ionic conductivity are the same for both networks, and thus the ohmic overpotential is notably higher for the extracted network at a fixed current density (0.09 V *vs.* 0.85 V at ∼70 mA cm^−2^ with the FTFF, not considering the membrane overpotential which is 0.13 V for both networks). The stark difference can be explained by the hydraulic transport through the networks. As the ionic conductivity is low for the TEMPO electrolyte, the hydraulic conductance of the networks becomes imperative to the electrode performance as it dictates the penetration of the reaction front from the membrane towards the current collector and is thus optimized for during the structure evolution (Fig. 5b). Due to the cubic structure with large pores and throats in the flow direction, but additionally in the through-plane direction causing a low resistance to flow, the ionic flux towards the membrane is high compared to the extracted network. The random orientation of the throats in the extracted network in combination with their small diameter, results in a low hydraulic conductance in the through-plane direction, and thus a high ohmic overpotential, resulting in lower species conversion compared to the cubic network (Fig. 5c, species conversion takes place predominantly near the membrane). The GA-PNM optimizes for the ionic conductance towards the membrane over the generations for both networks as is shown in Fig. A5b and A30,† resulting in a decrease in the ohmic overpotential of 13% (evaluated at ∼55 mA cm^−2^ for the extracted network and at ∼120 mA cm^−2^ for the cubic network, both with the FTFF). To this end the internal surface area near the membrane is increased in the cubic network to form throats with a large diameter, increasing the ionic conductance near the membrane to counterbalance the low ionic conductivity of the TEMPO electrolyte.

The 3x greater current output obtained with the cubic network compared to the extracted electrode at 0.5 V for the TEMPO electrolyte (even with surface area definition 1 for the cubic network which is an underestimation compared to definition 2) is substantial and should guide researchers for the design of next-generation electrodes for organic electrolytes where the ionic conductivity is generally the performance-limiting factor. However, a note must be made that the electrode dimensions and fluid flow rate most likely impact these results. Finally, we observed that with the TEMPO electrolyte, the trends for the IDFF between the extracted and cubic networks are similar compared to the vanadium electrolyte, suggesting that the influence of the flow field is not very different for the two investigated electrolytes at the evaluated electrode dimensions.

To conclude the chemistry-dependent optimization, we propose certain manufacturing guidelines for the fabrication of next-generation electrodes, specific to a given redox pair and electrolyte chemistry. For electrolytes with sluggish kinetics, electrodes with a high surface area are advantageous to decrease the activation overpotential. Where, especially for electrolytes with sluggish kinetics and high ionic conductivity, electrodes with numerous small pores and throats are beneficial, yet such structures result in a high pressure drop. Therefore, if this electrode structure would be combined with some in-plane electrolyte transport pathways along the flow direction, this would result in the best performance trade-off. Whereas for electrolytes with low ionic conductivity, electrodes with large throats in the through-plane direction (low through-plane tortuosity) result in increased performance. Especially for electrolytes with fast kinetics and low ionic conductivity, electrodes with large in- and through-plane electrolyte transport pathways result in increased current output and decreased pressure drop. However, the internal surface area of the electrodes should not be too low to be able to sustain a low activation overpotential.^40^ Furthermore, the presented guidelines for electrode design should go together with the selection of a suitable fabrication method and approach to translate the pore network to a solid structure as is often required for manufacturing purposes. While 3D printing is promising for optimized structures for electrolytes with low ionic conductivity and/or facile kinetics due to the high geometrical order that can be achieved, fabrication methods such as electrospinning or non-solvent induced phase separation might be more beneficial for kinetically-sluggish electrolytes as greater internal surface areas can be realized. Conventional manufacturing techniques to fabricate fibrous electrodes (*e.g.*, paper-making, hydroentangling, weaving), which are commercially used, do not offer the design space required to manufacture the geometrically controlled microstructures obtained in this work. Nonetheless, not only could these electrodes be manufactured with the optimized design by techniques such as 3D printing (by for example printing the solid part of the optimized pore networks^13^), but the learnings from the optimization (*e.g.*, bimodal electrodes with electrolyte transport pathways in the in- and through-plane directions) could also guide conventional electrode manufacturing towards enhanced electrodes for specific reactor architectures and operating conditions. Moreover, this work shows that the current GA-PNM framework can only optimize the performance of the electrodes to a certain degree. Consequently, with the current optimization approach, the selection of the starting network for the optimization is crucial. To diminish the importance of the starting network, mutation operators such as merging and splitting, or pore migration to induce freedom in the location of pores, should go together with the ability to change the number of pores, which could be investigated in depth in future work.

This work is a first step for the bottom-up optimization of electrode structures for flow electrochemical devices. By extending the presented tool, electrode optimization can be further improved and coupled to electrode manufacturing techniques. Possible extensions could include the incorporation of manufacturing constraints depending on the manufacturing technique of choice (*e.g.*, mechanical stability, shrinkage upon carbonization, converting to the solid structure), redefining the fitness function, adding more mutation parameters such as pore migration and changing the number of pores, and incorporating various pore and throat geometries (beyond spheres and cylinders) or refined geometrical definitions (*e.g.*, a better representation of the internal surface area). Other extensions could include reconsidering the network scaling step (as constraining the porosity limits the internal surface area enhancement), the optimization of larger electrode sizes, and optimizing the electrode and flow field designs in tandem. Finally, the translation to other electrochemical technologies, the coupling of the framework to computational fluid dynamic models (for higher detailed optimization or the simulation of advanced flow field geometries), and the full parallelization of the algorithm could be investigated. In conclusion, the optimization freedom in a genetic algorithm with many variables and constraints has both advantages and disadvantages. If the constraints are well understood and the variables are selected with care (*e.g.*, appropriate geometrical definitions), this method can be very powerful for the optimization of electrode structures from the bottom-up. If appropriate optimization conditions cannot be found, this approach can become inefficient where optimal solutions might not be obtained.^43^

## 4. Conclusions

In this work, we present a bottom-up tool for electrode optimization for tailored reactor architectures and operating conditions using the coupling of a genetic algorithm with a pore network model. The bottom-up approach was validated in our previous work and here we expanded the framework by adding pore merging and splitting functions, the ability to start the optimization from pore networks with a diverse morphology – including the optimization of benchmark materials – and the incorporation of the interdigitated flow field design. In addition, we elaborate on the possible choices in the optimization definitions including the selection of the objective function, geometrical definitions, network dimensions, and mutation and merging and splitting parameters. The genetic algorithm provides numerous optimization variables and constraints that must be well-understood and selected with care for successful optimization.

We found that mutation is the key operation driving the optimization, whereas pore merging and splitting can act as an additional mutation operation by inducing randomization and optimization beyond fixed pore coordinates. Moreover, the influence of system parameters, including the choice of electrolyte and flow field design, on the electrode optimization and performance is shown. For all analyzed systems, the genetic algorithm enhances the fitness by a strong reduction in pumping power of ∼51–77% and an improvement in electrical power of ∼8–39% by the formation of longitudinal flow pathways in the flow direction of large pores and throats with high hydraulic conductance, connected to regions with higher species conversion. We found that for the vanadium electrolyte, real electrodes extracted from X-ray tomographic images as starting structures speed up the electrode optimization, opening a path for the optimization of commercially available electrodes. Moreover, the structure evolution is strongly impacted by the flow field design because of the induced fluid path through the electrode. Furthermore, the electrode optimization was analyzed for two redox chemistries (VO^2+^/VO_2_^+^ and TEMPO/TEMPO^+^) for which substantial differences were observed in the current output with the cubic and extracted networks. For the vanadium chemistry with sluggish kinetics and high ionic conductivity, the extracted network results in the highest current output due to the presence of small pores with high mass transfer rates per unit volume, increasing the species conversion and thus the resulting current output. For the TEMPO electrolyte with fast kinetics and low ionic conductivity, electrodes with large in- and through-plane electrolyte transport pathways with low through-plane tortuosity have a higher current output because of the enhanced ionic conductance. Accordingly, we recommend that next-generation electrodes are optimized and manufactured tailored to the required reactor architectures and operating conditions.

In this study, we show the successful application of the genetic algorithm and that the obtained results can have a significant impact on the design of electrode structures for redox flow batteries. Hence, the developed tool can guide the design of next-generation electrodes for a broad range of operating conditions, electrolyte chemistries, reactor designs, and electrochemical technologies. Nevertheless, we encourage researchers to further extend the optimization framework by for example coupling the algorithm to suitable manufacturing techniques to further increase the impact and potential of this framework.

## Abbreviations

FTFF: Flow-through flow field
GA: Genetic algorithm
IDFF: Interdigitated flow field
PNM: Pore network model
RFB: Redox flow battery

## Symbol list

Δ*P*: Pressure drop, Pa
*A*_p_
: Pore internal surface area, m^2^
*A*_T_
: Throat internal surface area, m^2^
c
: Concentration, mol m^−3^
*c*_M_
: Mutation value
d
: Diameter, m
D
: Diffusion coefficient, m s^−1^
E
: Potential, V
I
: Generated total current, A
*j*_0_
: Exchange current density, A m^−2^
*L*_T_
: Throat length, m
*N*_T_
: Number of throats
P
: Power, W
Q
: Electrolyte flow rate, m^3^ s^−1^
*S*_p_
: Pore seed
*S*_T_
: Throat cross-sectional area, m^2^
u
: Electrolyte velocity, m s^−1^

### Greek

α: Charge transfer coefficient
*η*_p_
: Pumping energy efficiency
*μ*
: Viscosity, Pa s
*ξ*
: Fitness
*ρ*
: Density, kg m^−3^
*σ*
: Conductivity, S m^−1^
*σ*_M_
: Mutation range

### Superscripts

M: Mutated
o: Old

### Subscripts

1: Generation 1
1,2: Pore number, active species
a: Anodic
c: Cathodic
cell: Open circuit cell
el: Electrochemical
in: Inlet
losses: Losses
max: Thermodynamic maximum, maximum
n: Last generation
p: Pore
pump: Pumping
T: Throat

## Author contributions

M. v. d. H. contributed to the conceptualization, methodology, formal analysis, investigation, data curation, software, writing-original draft, writing-review and editing, and visualization. G. S. and V. d. H. contributed to methodology, software, and writing-review and editing. Finally, A. F. C. contributed to the conceptualization, methodology, funding, resources, writing-original draft, writing-review and editing, project administration, and supervision.

## Conflicts of interest

The authors declare no conflict of interest.

## Supplementary Material

DD-003-D3DD00247K-s001

## Data Availability

The code developed and expanded upon in this work can be found at https://github.com/MaximevdHeijden/GA-RFB-electrode/. The code runs with OpenPNM version 2.6. The data presented in this study can be provided by the corresponding author upon reasonable request and can be self-generated using our algorithms for which an example is provided in the repository. The parameters used for the runs discussed in this manuscript can be found in the Appendix (Section A9†).
