# Supplementary material for: A versatile optimization framework for porous electrode design
Source: Digit Discov. 2024 Apr 25;3(7):1292–307. doi: 10.1039/d3dd00247k (PMC11235177; doi:10.1039/d3dd00247k)
Supplement: DD-003-D3DD00247K-s001 [file DD-003-D3DD00247K-s001.pdf]

Electronic Supplementary Material (ESI) for Digital Discovery.
This journal is © The Royal Society of Chemistry 2024

Appendix

A versatile optimization framework for porous electrode design

Maxime van der Heijden,[a] Gabor Szendrei,[a] Victor de Haas,[a] and Antoni Forner-Cuenca[a*]

[a]Electrochemical Materials and Systems, Department of Chemical Engineering and Chemistry, Eindhoven University of Technology, PO Box 513, 5600 MB Eindhoven, Netherlands

** Corresponding author: a.forner.cuenca@tue.nl*

Author	ORCID number	Email address
Maxime van der Heijden	0000-0003-2250-4042	m.v.d.heijden@tue.nl
Gabor Szendrei		szendrei.gabor09@gmail.com
Victor de Haas		v.d.haas@student.tue.nl
Antoni Forner-Cuenca	0000-0002-7681-0435	a.forner.cuenca@tue.nl

Keywords: genetic algorithm, pore network model, porous electrode microstructure, flow field design, redox flow batteries, pore-scale modeling, electrochemical energy storage

A1. Network generation 2
A1.1. Artificial networks 2
A1.2. Extracted network 4
A2. Electrochemical algorithm 4
A2.1. Model equations 4
A2.2. Boundary conditions 5
A2.3. List of symbols 6
A3. Reference system 8
A4. Definitions of optimization functions 9
A4.1. Surface area definition 9
A4.2. Throat factor 12
A4.3. Electrode thickness 14
A4.4. Fitness function definition 14
A5. Beyond fixed lattice positions 18
A5.1. The effect of mutation 18
A5.2. The effect of merging and splitting 19
A6. Network evolution 21
A6.1. Extracted network 21
A6.2. Voronoi artificially generated network 22
A7. Impact of the flow field design 23
A8. The effect of the electrolyte chemistry 27
A9. Simulation parameters 31
A10. References 32

A1. Network generation

A1.1. Artificial networks

The cubic and Voronoi networks were artificially generated with the Cubic and Voronoi class algorithms in the OpenPNM Network module, respectively. The cubic lattice networks were created using a network shape, spacing, and connectivity as input parameters, where the shape of the lattices defined the number of pores in each dimension, the spacing the center-to-center distance between neighboring pores, and the connectivity the number of throats that were connected to every single pore. Furthermore, for this network, boundary pores with insignificant pore diameters (0.8 μm) were added to the surface of the network. The Voronoi networks, on the other hand, take the network shape and number of points as input parameters, where the network shape represents the size of the network and the number of points the amount of pores present in the chosen network size. For each base point, a convex polyhedron was defined, where the points were closer to the base point compared to the other points. Accordingly, the vertices and edges of these polyhedrals defined the pores and throats in the network, where the structure was framed by a cuboid, required to apply the boundary conditions to this network. For both networks, the geometrical properties of the pores and throats were attributed using the OpenPNM Geometry module, considering the geometry of spheres and cylinders.

For the initial cubic network structures, the pore diameter (d_p) was assigned by a pseudo-random process, depending on the maximum pore diameter ($d_{p,max}$) and a modification factor, the pore seed (S_p). The maximum pore diameter was determined by an iterative process by increasing the pore diameter until overlap with a neighboring pore was realized. The maximum pore diameter was subsequently multiplied with the pore seed, a value between 0.2 – 0.7, based on the standard option in the OpenPNM StickAndBall geometry class. The seed factor was pseudo-randomly generated for each pore to obtain the pore diameter:

$$d_p = S_p \cdot d_{p,max}. \qquad \text{(A1)}$$

The pore diameter for the initial Voronoi network, on the other hand, was defined by the maximum pore diameter and uniformly scaled down with the volume-scaling step. Moreover, for both networks, the throat diameter (d_T) between two pores was determined based on the pore diameters of the two connecting pores and a scaling factor, the throat factor. The throat diameter was based on the smallest pore size of the two connecting pores to prevent the formation of throats with a larger diameter than the connecting pore diameters. The smallest pore diameter was multiplied with the throat factor, which was fixed at 0.8 in this work based on the ratio of pore and throat diameters used by Sadeghi *et al.* to mimic the commercial SGL 25AA electrode used in flow batteries [1], to obtain the throat diameter:

$$d_T = 0.8 \cdot \min(d_{p,1}, d_{p,2}). \qquad \text{(A2)}$$

As the throats are represented as cylinders, the throat cross-sectional area (S_T) was described by the throat diameter by **equation (A3)** and the throat internal surface area (A_T) by the throat diameter and length (L_T), defined as the length between two throat endpoints,

according to **equation (A4)**. The throat conduit length, which is equal to the network spacing for cubic networks, was used for the hydraulic transport through the network and was described by the summation of the length of two half-pores and the length of the connected throat.

$$S_T = \frac{\pi}{4} \cdot d_T^2 \tag{A3}$$

$$A_T = \pi \cdot d_T \cdot L_T \tag{A4}$$

Finally, the initial definition of the pore internal surface area (A_p), described by the OpenPNM geometry functions, was defined by subtracting the throat cross-sectional area of N_T number of neighboring throats from the pore surface area. The definition is a simplification of the pore internal surface area as the curvature of the intersection between the pore and throat was not considered.

$$A_p = \pi \cdot d_p^2 - \sum_{j=1}^{N_T} S_T \qquad \text{Definition 1} \tag{A5}$$

Because of the direct correlation between surface area and electrochemical performance, multiple surface area definitions were considered in this work, see **equations (A5-A8)**. All considered definitions are simplified descriptions of the surface area, identified to study the influence of geometrical definitions on the structure evolution.

$$A_p = \pi \cdot d_p^2 \qquad \text{Definition 2} \tag{A6}$$

$$A_p = \pi \cdot d_p^2 - \sum_{j=1}^{N_T} (0.8 \cdot d_p)^2 \cdot \frac{\pi}{4} \qquad \text{Definition 3} \tag{A7}$$

$$A_p = \pi \cdot d_p^2 - \sum_{j=1}^{N_T} S_T + \sum_{j=1}^{N_T} 0.5 \cdot A_T \qquad \text{Definition 4} \tag{A8}$$

The network properties of the cubic and Voronoi structures are given in **Table A1**, where, in this work, small three-dimensional electrode structures were optimized with electrode geometrical areas of approximately 500 x 500 μm^2. To allow comparison between the three network structures, the network shape and spacing were based on the microstructural properties of the off-the-shelf Freudenberg H23 paper electrode (Fuel Cell Store, 80 % porosity) with a median pore size of 20 μm and a measured thickness of 210 μm [2–5]. As the network porosity (summation of the total pore and throat volumes) and the number of pores and throats are a direct result of the chosen input parameters, to maintain a constant porosity between the three structures, the reference network of the Voronoi structure was chosen to have a diameter of 25 μm (additionally used for the reference network) and 300 points as input parameters.

Table A1: Network properties used for the optimization study for the cubic, Voronoi, and extracted Freudenberg H23 electrode.

Parameter	Cubic	Voronoi	Extracted	Unit
Porosity	54	49	51	%
Network shape	[13, 13, 4]	-	-	-
Number of pores	676	1755	3348	-
Number of throats	1755	3506	10171	-
Network dimensions	580 x 580 x 220	500 x 500 x 220	500 x 500 x 198	μm^3
Pore seed	$0.2<S_p<0.7$	-	-	-
Spacing	40	-	-	μm
Connectivity	6	-	-	-

A1.2. Extracted network

The extracted network used in this study was the Freudenberg H23 carbon paper electrode, imaged in a previous study [4] with X-ray computed tomography. After image processing including a two-dimensional median filter and a K-means cluster segmentation filter, the pore network was extracted using the SNOW algorithm that makes use of a marker-based watershed segmentation algorithm [6], using the inscribed pore diameter. The extraction was performed on a single Intel® Core™ i7-8750H CPU. During the extraction, the geometrical properties of the pores and throats within this network were automatically assigned considering the geometry of spheres and cylinders; however, to match with the geometrical definitions used in the GA, the network geometry was scaled to the definitions described in **equations (A2-A4** and **A6)**. The internal surface area definition of **equation (A6)** was used because with a connectivity >6, the large throat cross-sectional area subtraction resulted in a negative internal surface area and consequently the optimization of small pores.

A2. Electrochemical algorithm

A2.1. Model equations

In the PNM, the fluid transport can be independently solved from the mass and charge transport because of the dilute electrolyte assumption [1]. Hence, the fluid transport was first solved to obtain the pressure field with the Navier-Stokes equation. Using the Hagen-Poiseuille equation, the velocity field was subsequently back-calculated from the pressure field. The solved pressure field was then fed into the iterative algorithm to solve the coupled mass and charge transport equations with the advection-diffusion-reaction and the conservation of charge equations, coupled by the Butler-Volmer equation to obtain the species concentration and potential fields. Furthermore, the coupling between the anodic and cathodic compartments was established at the membrane by treating the membrane as a macro continuum entity by only considering the overall macroscopic ionic resistance of the membrane with Ohm’s law. This allowed for the coupling of the charge transport within one compartment with the electrolyte potential at the membrane boundary in the other compartment.

The PNM was solved at a constant applied arbitrary overpotential of -0.5 V in discharge mode, with an initial guess for the concentration and overpotential fields. The initial guess for

the concentration field was equal to the inlet concentration for all networks (100 mol m^{-3} for all redox species). The initial guess of the overpotential was updated after solving for each network to speed up the iterative approach, where the initial guess for the first network in the first generation was set to ±0.25 V. In the iterative approach, two numerical strategies were employed to counteract the solution divergence as a result of the high nonlinear nature of the system. The first strategy was imposing an under-relaxation scheme on the concentration and potential fields with a constant relaxation factor, and the second strategy was the linearization of the charge transport source term [4]. Finally, numerical convergence was achieved when both the relative and absolute tolerances were met for the total current, bound by a maximum number of iterations [4].

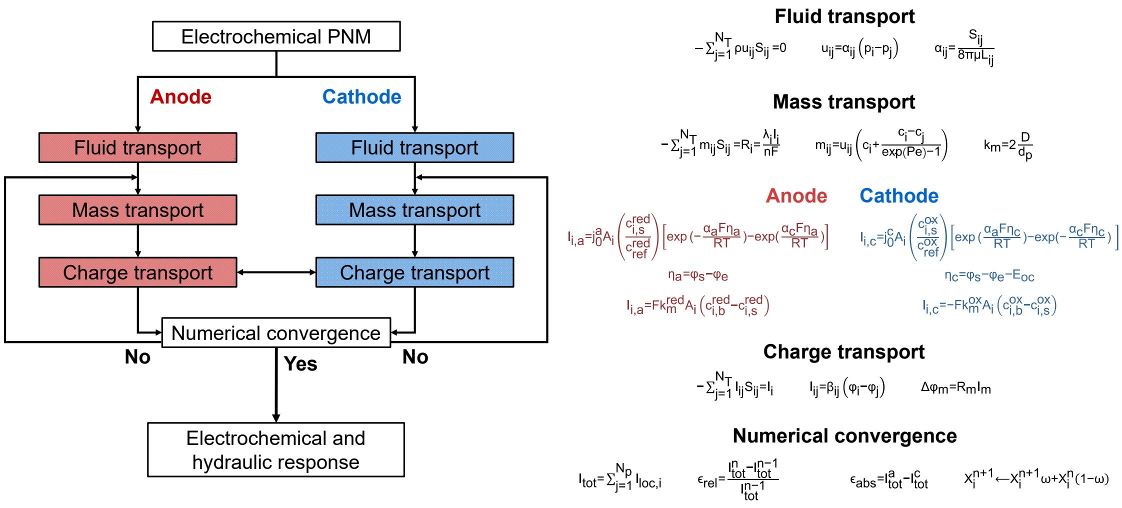

Figure A1: Flowchart and model equations of the pore network model incorporated into the genetic algorithm. More information regarding the pore network model physics can be found in [4].

A2.2. Boundary conditions

Because of symmetry along the width for the FTFF, the modeled domain consisted of two symmetric, mirrored ~0.5 x 0.5 mm^2 electrodes. For the IDFF, the modeling domain was chosen to be half an inlet channel, a full rib, and half an outlet channel. We assumed the channel and rib dimensions to be 0.5 mm wide, resulting in ~1 x 0.5 mm^2 electrodes for the IDFF. The boundary conditions for the individual flow fields are shown in **Figure A2**.

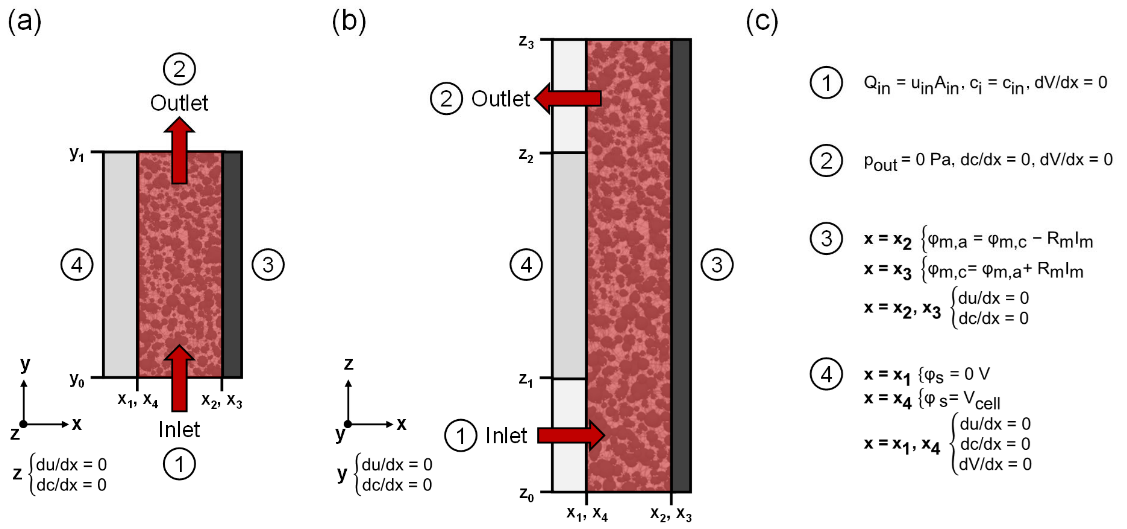

Figure A2: Schematic representation of the boundary conditions applied in the electrochemical pore network model for one representative model element and one half cell of the symmetric flow battery. x_1-x_4 represents the electrode thickness, 0-y_1 the length, and 0-z_3 the width coordinates of the different interfaces, with (1) the flow field inlet, (2) flow field outlet, (3) membrane, and (4) current collector, shown for: **(a)** the flow-through flow field, and **(b)** the interdigitated flow field. **(c)** The boundary conditions applied for the different interfaces.

A2.3. List of symbols

Symbol	Description	Units
A_i	Electrochemically active internal surface area of pore i	m^2
A_{in}	Geometrical inlet area of the electrode	m^2
c	Concentration	mol m^{-3}
D	Diffusion coefficient	m^2 s^{-1}
d_p	Pore diameter	m
E_{oc}	Open-circuit potential	V
F	Faradaic constant, 96,485 C mol^{-1}	C mol^{-1}
I	Current	A
I_{ij}	Charge flux	A m^{-2}
j_0	Exchange current density	A m^{-2}
k_m	Mass transfer coefficient	m s^{-1}
L	Conduit length, length of the medium	m
m	Mole flux	mol m^{-2} s^{-1}
n	Number of electrons, or iteration number	-
N_p	Number of pores	-
N_T	Number of throats	-
p	Pressure	Pa
Pe	Peclet number	-
Q	Flow rate	m^3 s^{-1}
R	(a) Molar gas constant, 8.314 J $mol^{-1}K^{-1}$	J mol^{-1} K^{-1}
	(b) Resistance	Ω
	(c) Net reaction rate	mol s^{-1}
S	Cross-sectional area	m^2

T	Operating temperature	K
u	Fluid velocity	m s^{-1}
V	Voltage	V
V_{cell}	Given cell potential	V
X	Concentration or potential	mol m^{-3} or V
	**Greek**	
α	(a) Transfer coefficient	-
	(b) Hydraulic conductance of the throat	m Pa^{-1} s^{-1}
β	Electrical conductance	S m^{-2}
ϵ	Error value	-
η	Overpotential	V
λ	Stoichiometric coefficient	-
μ	Dynamic viscosity	Pa s
ρ	Electrolyte density	kg m^{-3}
φ	Potential	V
ω	Relaxation factor	-
	**Superscripts**	
a	Anode	
c	Cathode	
n	Iteration number	
ox	Oxidized form	
red	Reduced form	
	**Subscripts**	
a	Anode	
abs	Absolute	
b	Bulk	
c	Cathode	
e	Electrolyte, liquid phase	
i	Within pore i, species i	
ij	Pore i to pore j, throat ij	
in	Inlet	
j	Within pore j, species j	
loc	Local	
m	Membrane	
ref	Reference	
rel	Relative	
s	Solid phase, surface	
tot	Total	

A3. Reference system

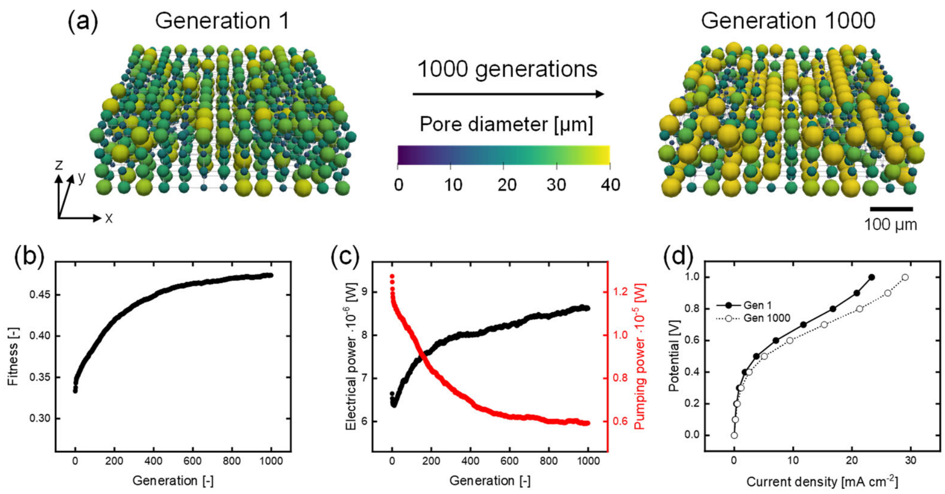

Figure A3: The results of the genetic algorithm optimized for the VO^{2+}/VO_2^+ electrolyte, evaluated for the **reference** system (cubic network with mutation and a flow-through flow field), with: **(a)** the structure evolution over 1000 generations with the flow in the y-direction and the thickness in the z-direction with the membrane facing to the top, **(b)** the maximum fitness evolution, **(c)** the maximum electrical power and pumping power evolution, and **(d)** the polarization curve for the first and last generation.

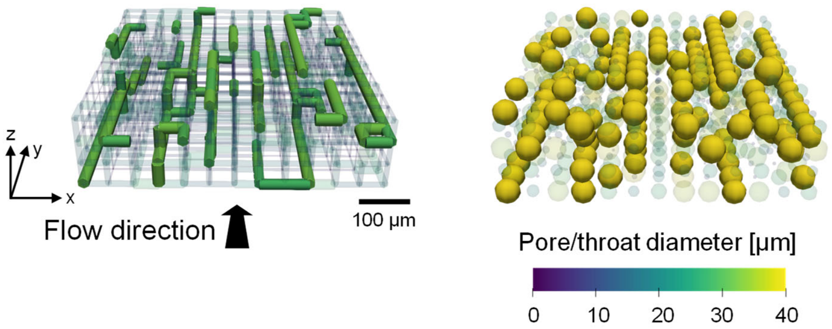

Figure A4: Visualization of the longitudinal transport pathways formed during the optimization of the reference network (**Figure A3**), with on the left side the throats of the optimized structure where the throats > 28 µm are highlight, and on the right side the pores of the optimized structure with the pores >39 µm highlighted.

A4. Definitions of optimization functions

A4.1. Surface area definition

The *internal surface area* is complex to define as the translation of a macroporous electrode (e.g., fibrous mat) to a pore network based on spheres and cylinders is a simplification of the complex porous space and does not consider the surface roughness or electrochemical activity of the surface. Therefore, the simplified internal surface area definition used in the PNM should be based on the type of electrode structure and manufacturing technique. For example, OpenPNM defines the internal surface area by **equation (A5)**, which results in that small pores have a relatively smaller surface area per unit volume, compared to larger pores, because of the larger relative subtraction for the throat cross-sectional areas. With this definition and the fixed lattice positions where each pore can occupy a fixed volume, large pores are beneficial as they both reduce the pressure drop and increase the surface area (larger surface area per constant volume), especially when connected to small pores (small subtraction of the throat cross-sectional areas, **equation (A5)**). However, when not bound to fixed pore coordinates with a fixed volume, smaller pores should be beneficial for a high surface area per unit volume, whereas large interconnected pores mostly dictate the electrolyte transport [2–4,7,8]. To investigate the effect of the internal surface area definition on the structure evolution, four surface area definitions (**equations (A5-A8)**) were introduced in **Section A1.1**.

We observed that by changing this geometry parameter definition, the electrode optimization is affected, even for the cubic network with fixed pore coordinates (**Figures A5-A7** and **Tables A2-A5**). First, the internal surface area of the electrodes is altered, impacting the electrochemical power directly and thus the fitness value of the first generation. The surface area increase is significantly higher when using definition 1, while it is even decreased when using definition 4. For definitions 1 - 3, the surface area is enhanced when the transport pathways consisting of large pores are formed, as the throat cross-sectional area subtraction is small in definitions 1 and 3, and large pores have a high surface area in definition 2, whereas the formation of these pathways is unfavorable with definition 4. The effect on the electrical power can also be seen in the surface area distribution of the individual electrode layers (no significant increase near the membrane for definitions 2 - 4) and the pumping power decrease (more significant for definitions 2 - 4), shifting the optimization towards pumping power optimization for definitions 2 - 4. Nevertheless, we find that all surface area definitions result in optimized structures with a bimodal pore size distribution with longitudinal transport pathways in the flow direction that reduce the pumping power.

The comparison between definition 1 and definition 2 was also made with the extracted network as definition 1 resulted in a negative internal surface area and therefore not in the formation of longitudinal transport pathways in the flow direction (**Figure A8**). When applying definition 2, these pathways are formed as expected (**Figure A9**). The internal surface area obtained using **equation (2)** only results in a underestimation of the internal surface area in comparison with literature values (i.e., 2.2 x 10^4 m^{-1} using **equation (2)** *vs.* 7.2 x 10^4 m^{-1} obtained in the literature for the Freudenberg H23 electrode [3,4]). This makes us believe that for this computational work, **equation (2)** is a reasonable assumption, considering the simplification of the geometrical properties with PNMs, of the surface area for the extracted

electrode with a high connectivity. Hence, the cubic and extracted electrodes are optimized with distinct internal surface area definitions and therefore the optimization trends should mainly be compared rather than assessing the optimization results quantitively.

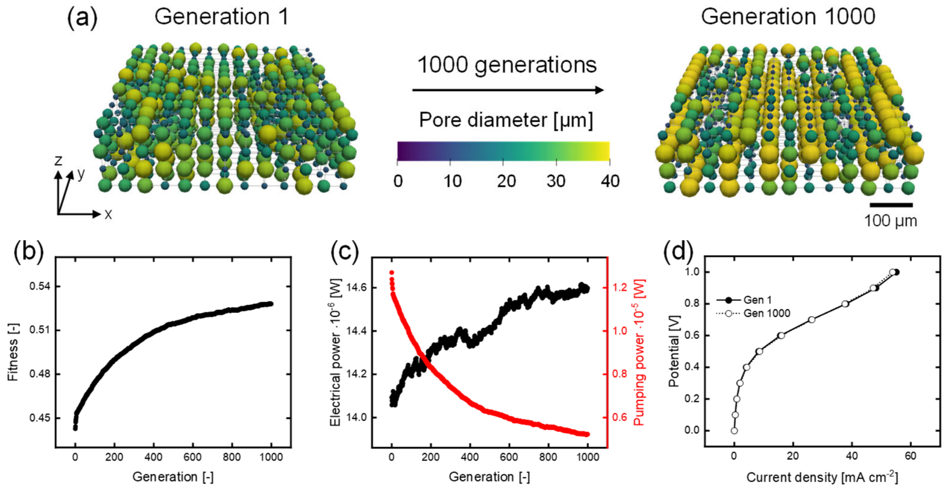

Figure A5: The results of the genetic algorithm optimized for the VO^{2+}/VO_2^+ electrolyte, evaluated for the reference network with surface area **definition 2**, with: **(a)** the structure evolution over 1000 generations with the flow in the y-direction and the thickness in the z-direction with the membrane facing to the top, **(b)** the maximum fitness evolution, **(c)** the maximum electrical power and pumping power evolution, and **(d)** the polarization curve for the first and last generation.

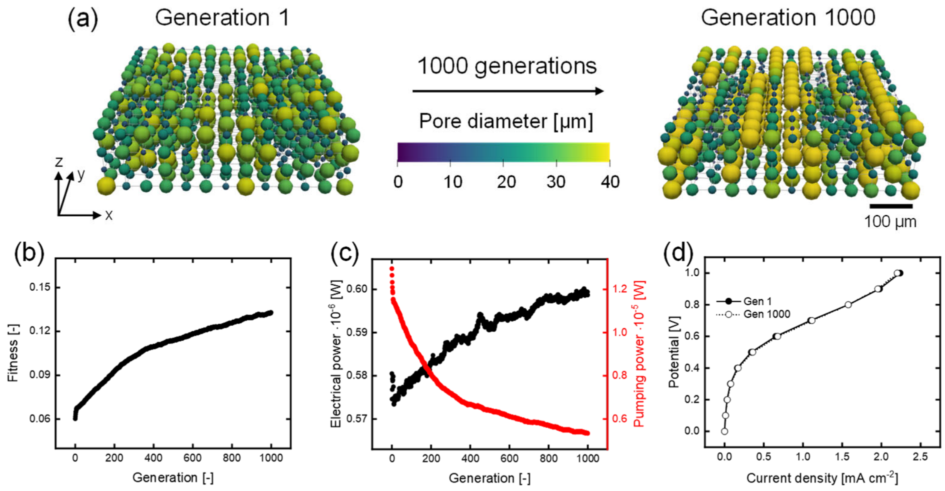

Figure A6: The results of the genetic algorithm optimized for the VO^{2+}/VO_2^+ electrolyte, evaluated for the reference network with surface area **definition 3**, with: **(a)** the structure evolution over 1000 generations with the flow in the y-direction and the thickness in the z-direction with the membrane facing to the top, **(b)** the maximum fitness evolution, **(c)** the maximum electrical power and pumping power evolution, and **(d)** the polarization curve for the first and last generation.

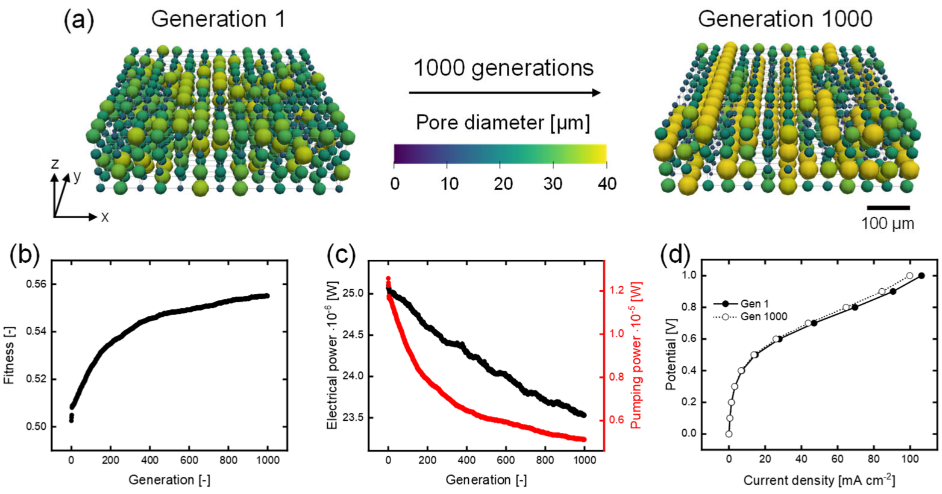

Figure A7: The results of the genetic algorithm optimized for the VO^{2+}/VO_2^+ electrolyte, evaluated for the reference network with surface area **definition 4**, with: **(a)** the structure evolution over 1000 generations with the flow in the y-direction and the thickness in the z-direction with the membrane facing to the top, **(b)** the maximum fitness evolution, **(c)** the maximum electrical power and pumping power evolution, and **(d)** the polarization curve for the first and last generation.

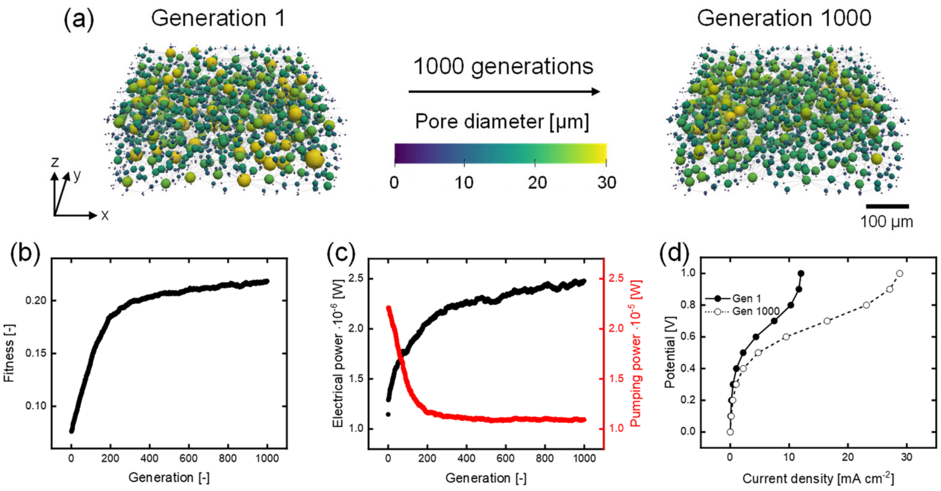

Figure A8: The results of the genetic algorithm optimized for the VO^{2+}/VO_2^+ electrolyte, evaluated for the **extracted Freudenberg H23 electrode** (with mutation and a flow-through flow field) with surface area definition 1, with: **(a)** the structure evolution over 1000 generations with the flow in the y-direction and the thickness in the z-direction with the membrane facing to the top, **(b)** the maximum fitness evolution, **(c)** the maximum electrical power and pumping power evolution, and **(d)** the polarization curve for the first and last generation.

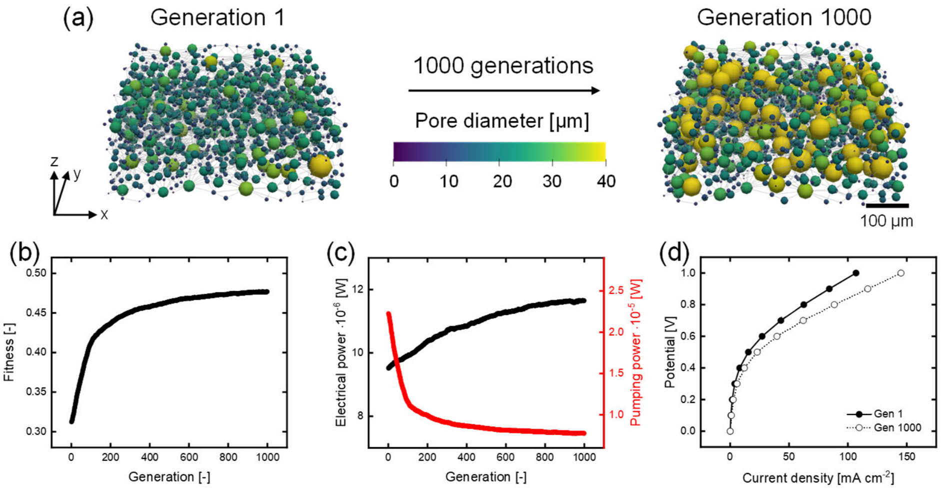

Figure A9: The results of the genetic algorithm optimized for the VO^{2+}/VO_2^+ electrolyte, evaluated for the extracted Freudenberg H23 electrode (with mutation and a flow-through flow field) with surface area **definition 2**, with: **(a)** the structure evolution over 1000 generations with the flow in the y-direction and the thickness in the z-direction with the membrane facing to the top, **(b)** the maximum fitness evolution, **(c)** the maximum electrical power and pumping power evolution, and **(d)** the polarization curve for the first and last generation.

A4.2. Throat factor

Moreover, the *geometrical definitions* used in the algorithm, e.g., the definitions of OpenPNM (**equations (A1-A5)**), strongly affect the optimization as they directly impact the electrochemical and pumping power. For example, the *throat factor* (**equation (A2)**) chosen in this work as 0.8 [1], affects the pressure drop and internal surface area of the network, both by the throat cross-sectional area. The impact of the throat diameter on the structure evolution was analyzed by adjusting the throat factor to 0.7 and 0.9:

$$d_T = 0.7 \cdot \min\left(d_{p,1}, d_{p,2}\right) \tag{A9}$$

$$d_T = 0.9 \cdot \min\left(d_{p,1}, d_{p,2}\right), \tag{A10}$$

with d_T the throat diameter and d_p the pore diameter of the connecting pores. Changing the the throat diameter affects the throat size relative to the pore size and thus directly alters the pressure drop. Additionally, the internal surface area and thus the electrochemical performance changed more drastically (8 % increase in electrochemical performance for a factor of 0.7 and 102 % for a factor of 0.9, compared to 30 % for the reference system). Moreover, by applying the OpenPNM geometry definitions described in **Section A1** to *the extracted network*, the internal surface area is significantly impacted and decreased by ~30 x (from a total internal surface area of 6.0 x 10^5 m^{-1} to 2.2 x 10^4 m^{-1}), strongly decreasing the current output of the electrode (at 0.5 V: 15 mA cm^{-2} *vs.* 108 mA cm^{-2}, and at 1 V: 107 mA cm^{-2} *vs.* 726 mA cm^{-2}). This shows the high sensitivity of the system (stochastic nature and the sensitivity to the geometrical definitions), which should be handled and controlled with care, e.g., through a volume scaling step, to allow for a fair comparison between optimization cases.

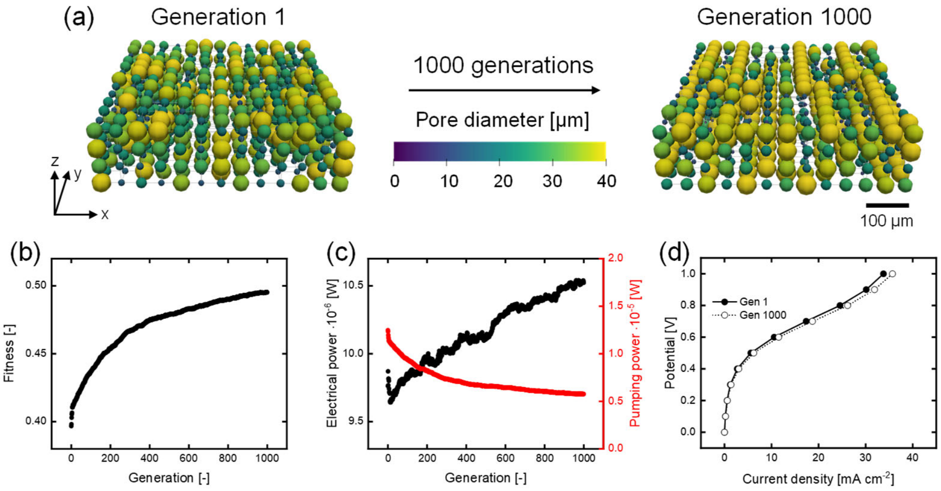

Figure A10: The results of the genetic algorithm optimized for the VO^{2+}/VO_2^+ electrolyte, evaluated for the reference system (cubic network with mutation and a flow-through flow field), with a **throat factor of 0.7**, with: **(a)** the structure evolution over 1000 generations with the flow in the y-direction and the thickness in the z-direction with the membrane facing to the top, **(b)** the maximum fitness evolution, **(c)** the maximum electrical power and pumping power evolution, and **(d)** the polarization curve for the first and last generation.

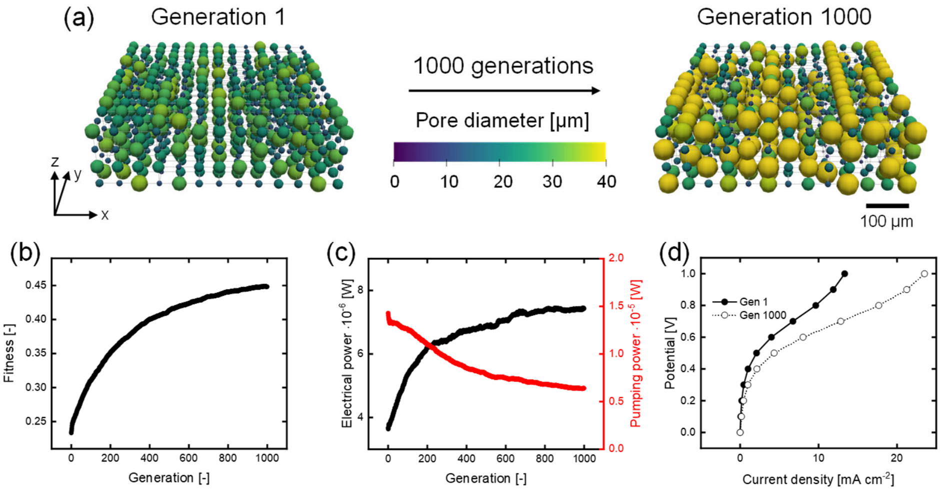

Figure A11: The results of the genetic algorithm optimized for the VO^{2+}/VO_2^+ electrolyte, evaluated for the reference system (cubic network with mutation and a flow-through flow field), with a **throat factor of 0.9**, with: **(a)** the structure evolution over 1000 generations with the flow in the y-direction and the thickness in the z-direction with the membrane facing to the top, **(b)** the maximum fitness evolution, **(c)** the maximum electrical power and pumping power evolution, and **(d)** the polarization curve for the first and last generation.

A4.3. Electrode thickness

Moreover, an *electrode size* that provides a representative elementary volume of a manufacturable electrode must be utilized. In this study, we arbitrarily chose a ~500 x 500 x 200 μm^3 electrode for the FTFF and a ~1000 x 500 x 200 μm^3 electrode for the IDFF. To show the effect of the electrode dimensions, we show the optimization for an electrode that is twice as thick in **Figure A12**.

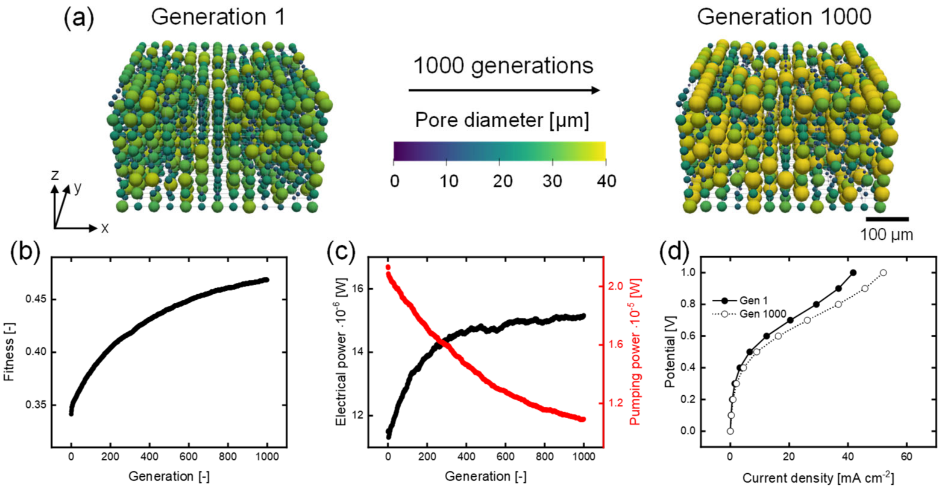

Figure A12: The results of the genetic algorithm optimized for the VO^{2+}/VO_2^+ electrolyte, evaluated for the reference system with an electrode **thickness of 400 µm**, with: **(a)** the structure evolution over 1000 generations with the flow in the y-direction and the thickness in the z-direction with the membrane facing to the top, **(b)** the maximum fitness evolution, **(c)** the maximum electrical power and pumping power evolution, and **(d)** the polarization curve for the first and last generation.

Furthermore, the network scaling step could be reconsidered as constraining the porosity also limits the internal surface area enhancement. Finally, further factors that need to be selected before optimization are the parent-individual ratio and the mutation probability and range. These factors impact the optimization time required and the number of generations necessary for the optimization. The optimization freedom in a genetic algorithm with many variables and constraints has both advantages and disadvantages. If the constraints are well-understood and the variables are selected with care, this method can be very powerful for the optimization of electrode structures from the bottom-up. If appropriate optimization conditions cannot be found, this approach can become inefficient where optimal solutions might not be obtained [9].

A4.4. Fitness function definition

The objective of the GA is to optimize electrode structures based on a defined fitness function. Therefore, first, a suitable *fitness function* needs to be determined that considers the objective of the optimization. In this work, we use the definition of **equation (3)**, in which the pressure drop and current density of the networks have equal weight. **Equation (3)** optimizes to a maximum fitness of ξ = 0.603, which is achieved when the pumping power is zero. Thus, networks with a large starting fitness can only have a small relative fitness increase, though the percentual improvement in electrical and pumping power is absolute. Here, the effect of the

fitness function on the optimization is investigated by performing optimization studies with only the pumping power or only the electrochemical power considered, according to:

$$\xi_{pump} = \frac{P_{max}}{P_{max} + P_{pump}} \tag{A11}$$

$$\xi_{el} = P_{max} - P_{el}. \tag{A12}$$

With ξ the fitness function, P_{max} the thermodynamic maximum electrochemical power, P_{pump} the pumping power, and P_{el} the electrochemical power loss. From this study, we find that when only optimizing for the *electrochemical power*, the pumping power remains unoptimized, even resulting in a stark increase of 721 % in the required pumping power. On the contrary, the electrochemical power improves significantly by 101 % because of the formation of large pores with a maximum pore diameter (38 - 40 µm) connected to small pores (<10 µm), increasing the internal surface area of the electrode according to **equation (A5)**. The resulting network (**Figure A14**) is drastically different compared to the reference network (**Figure A3**) as no longitudinal transport pathways are formed in the flow direction but rather alternating large and small pores connected by throats with a small diameter (resulting in the increase in pumping power) [5]. Moreover, the resulting pore and throat size distributions are different, see **Figure A15**, where significantly more pores are formed of 40 µm (164 *vs.* 66), accompanied by more small throats of <10 µm (1290 *vs.* 800) compared to the reference fitness function. On the other hand, when optimizing for the *pumping power* alone, the optimization is rather comparable to that of the reference network, additionally with an increase in electrochemical power (34 %). Hence, suggesting that the original fitness definition, with equal weight, steers towards the optimization of the pumping power over the electrochemical power. The presented study shows the importance of selecting a suitable fitness definition for the electrode optimization.

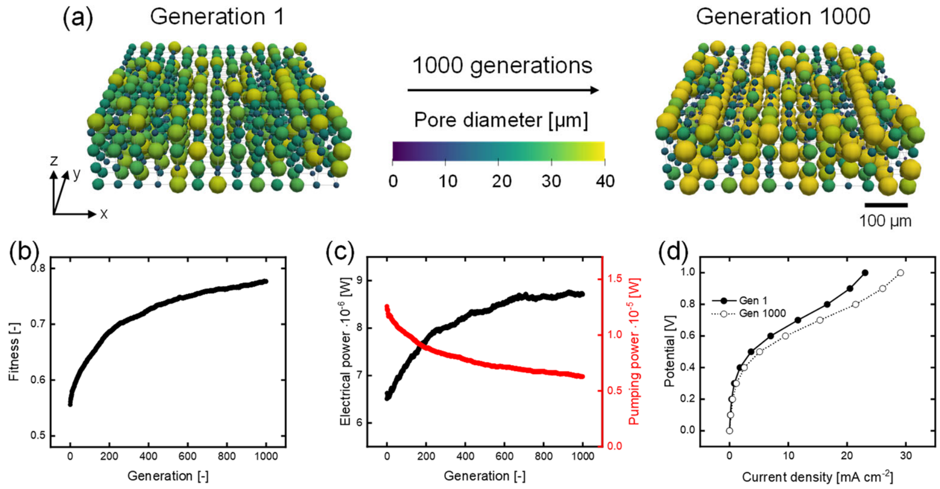

Figure A13: The results of the genetic algorithm optimized for the VO^{2+}/VO_2^+ electrolyte, evaluated for the reference system (cubic network with mutation and a flow-through flow field), and optimized for only the **pumping power**, with: **(a)** the structure evolution over 1000 generations with the flow in the y-direction and the thickness in the z-direction with the membrane facing to the top, **(b)** the maximum fitness evolution, **(c)** the maximum electrical power and pumping power evolution, and **(d)** the polarization curve for the first and last generation.

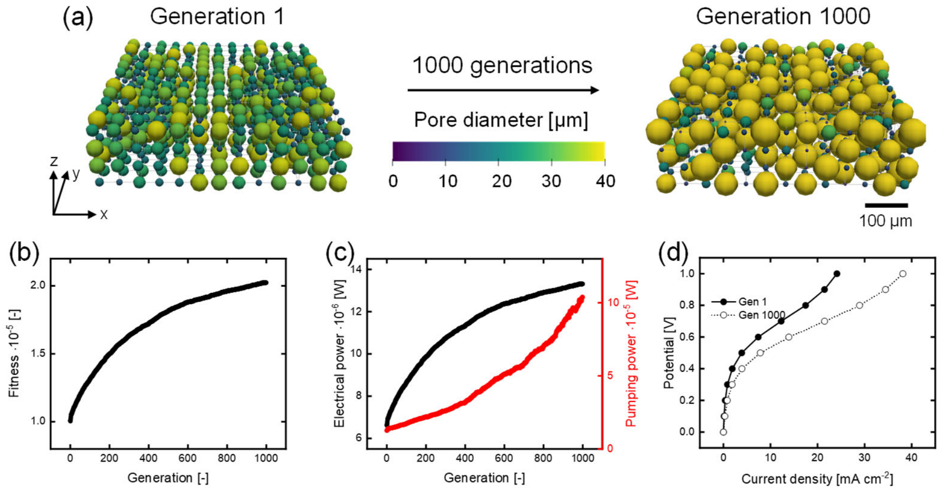

Figure A14: The results of the genetic algorithm optimized for the VO^{2+}/VO_2^+ electrolyte, evaluated for the reference system (cubic network with mutation and a flow-through flow field), and optimized for only the **electrochemical power**, with: **(a)** the structure evolution over 1000 generations with the flow in the y-direction and the thickness in the z-direction with the membrane facing to the top, **(b)** the maximum fitness evolution, **(c)** the maximum electrical power and pumping power evolution, and **(d)** the polarization curve for the first and last generation.

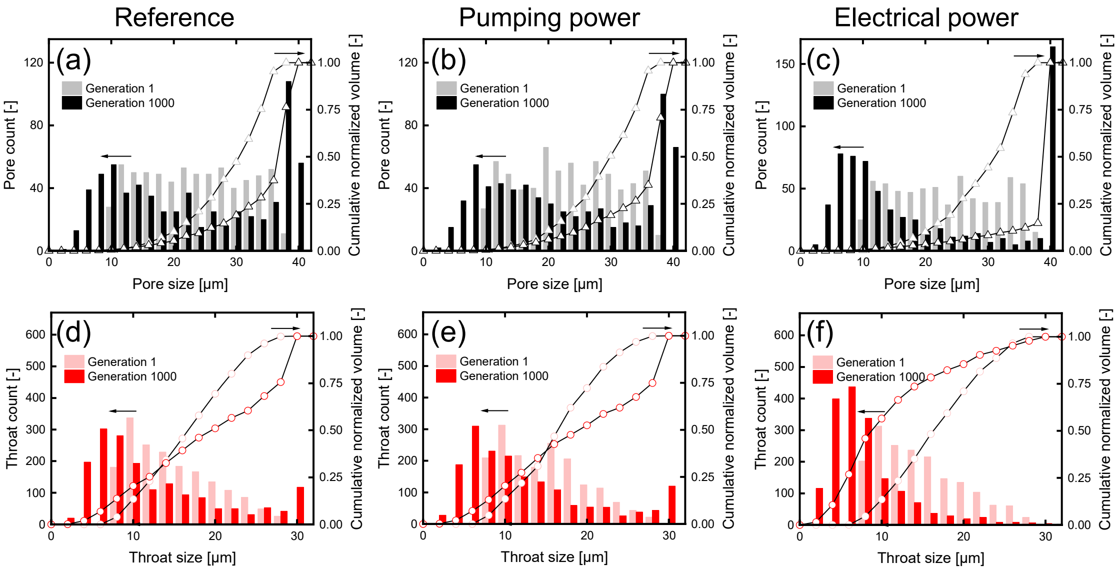

Figure A15: The **(a-c)** pore and **(d-f)** throat size distributions of the first and final generation, showing the pore or throat count and cumulative normalized volume distributions (divided in 2 μm pore or throat sized bins) for **(a)** the reference system, **(b)** optimized for only the pumping power, and **(c)** optimized for only the electrical power.

Table A2: The absolute values of the fitness and surface area for the best-performing network in the first and final generation for the definitions of optimization functions comparisons.

	**Fitness [-]**		**Surface area [m^2]**	
	**Gen 1**	**Gen 1000**	**Gen 1**	**Gen 1000**
Reference (Definition 1)	0.33	0.47	6.8 x 10^{-7}	9.0 x 10^{-7}
Definition 2	0.44	0.53	1.4 x 10^{-6}	1.5 x 10^{-6}
Definition 3	0.06	0.13	5.8 x 10^{-8}	6.1 x 10^{-8}
Definition 4	0.50	0.56	2.6 x 10^{-6}	2.4 x 10^{-6}
Definition 1 – extracted	0.05	0.22	-1.1 x 10^{-7}	-2.3 x 10^{-7}
Definition 2 – extracted	0.31	0.48	1.1 x 10^{-6}	1.4 x 10^{-6}
Throat factor 0.7	0.40	0.50	1.0 x 10^{-6}	1.1 x 10^{-6}
Throat factor 0.9	0.23	0.45	3.7 x 10^{-7}	7.9 x 10^{-7}
400 μm in thickness	0.34	0.47	1.2 x 10^{-6}	1.6 x 10^{-6}
Pumping power only	0.56	0.78	6.6 x 10^{-7}	9.1 x 10^{-7}
Electrochemical power only	1.0 x 10^{-5}	2.0 x 10^{-5}	6.7 x 10^{-7}	1.5 x 10^{-6}

Table A3: The absolute values of the electrical power and pumping power for the best-performing network in the first and final generation for the definitions of optimization functions comparisons.

	**Electrical power [W]**		**Pumping power [W]**	
	**Gen 1**	**Gen 1000**	**Gen 1**	**Gen 1000**
Reference (Definition 1)	6.5 x 10^{-6}	8.5 x 10^{-6}	1.2 x 10^{-5}	5.9 x 10^{-6}
Definition 2	1.4 x 10^{-5}	1.5 x 10^{-5}	1.2 x 10^{-5}	5.2 x 10^{-6}
Definition 3	6.0 x 10^{-7}	6.0 x 10^{-7}	1.3 x 10^{-5}	5.3 x 10^{-6}
Definition 4	2.5 x 10^{-5}	2.4 x 10^{-5}	1.2 x 10^{-5}	5.1 x 10^{-6}
Definition 1 – extracted	1.2 x 10^{-6}	2.5 x 10^{-6}	3.2 x 10^{-5}	1.1 x 10^{-5}
Definition 2 – extracted	1.0 x 10^{-5}	1.2 x 10^{-5}	2.2 x 10^{-5}	7.8 x 10^{-6}
Throat factor 0.7	1.0 x 10^{-5}	1.1 x 10^{-5}	1.2 x 10^{-5}	5.8 x 10^{-6}
Throat factor 0.9	3.7 x 10^{-6}	7.5 x 10^{-6}	1.4 x 10^{-5}	6.4 x 10^{-6}
400 μm in thickness	1.2 x 10^{-5}	1.5 x 10^{-5}	2.1 x 10^{-5}	1.1 x 10^{-5}
Pumping power only	6.5 x 10^{-6}	8.5 x 10^{-6}	1.2 x 10^{-5}	6.3 x 10^{-6}
Electrochemical power only	6.5 x 10^{-6}	1.4 x 10^{-5}	1.2 x 10^{-5}	1.0 x 10^{-4}

Table A4: Percentual increase in fitness, electrical power, and pumping power over 1000 generations for the best-performing network in all evaluated systems for the definitions of optimization functions comparisons.

**Percentual increase [%]**	**Fitness**	**Electrical power**	**Pumping power**	**Surface area**
Reference (Definition 1)	42	30	-54	34
Definition 2	19	3.6	-59	5.6
Definition 3	121	3.1	-59	6.3
Definition 4	10	-6.1	-59	-5.4
Definition 1 – extracted	346	116	-67	119
Definition 2 – extracted	53	22	-65	31
Throat factor 0.7	25	7.8	-54	9.2
Throat factor 0.9	92	102	-55	115
400 μm in thickness	37	32	-49	38
Pumping power only	40	34	-50	38
Electrochemical power only	101	101	721	119

Table A5: Total pore surface area values for each network layer for the best-performing network in the first and final generation from the current collector to the membrane, evaluated for the definitions of optimization functions comparisons.

Pore surface area x 10^{-7} [m^2]	Generation	Current collector	Internal 1	Internal 2	Membrane
Reference (Definition 1)	1	1.9	1.5	1.3	1.8
	1000	2.5	1.8	2.2	2.4
Definition 2	1	3.4	3.5	3.6	3.7
	1000	3.4	3.8	4.4	3.5
Definition 3	1	1.5	1.4	1.4	1.4
	1000	1.7	1.3	1.7	1.5
Definition 4	1	6.2	6.7	6.6	5.9
	1000	6.2	5.9	6.3	5.7
Throat factor 0.7	1	2.4	2.3	2.4	2.7
	1000	2.8	2.5	2.7	2.9
Throat factor 0.9	1	1.1	0.73	0.74	1.0
	1000	2.2	1.7	1.8	2.2
Pumping power only	1	1.8	1.3	1.5	1.7
	1000	2.7	1.7	2.2	2.5
Electrochemical power only	1	1.9	1.5	1.5	1.9
	1000	3.8	3.2	3.6	4.2

A5. Beyond fixed lattice positions

A5.1. The effect of mutation

The effect of *mutation* was investigated by running the GA with merging and splitting (0.1 % chance per pore per generation) with and without mutation. Moreover, the genetic algorithm without any form of mutation (no mutation or merging and splitting) was analyzed and the results are shown in **Tables A6 - A8**.

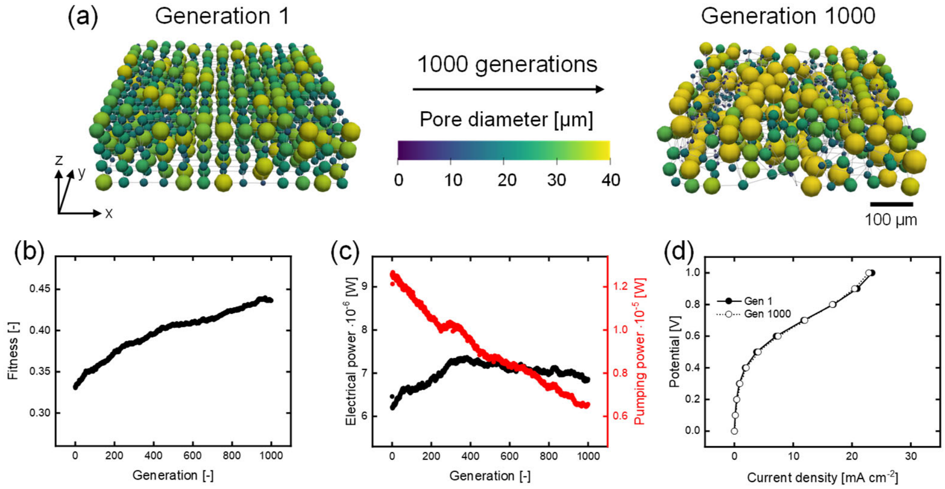

Figure A16: The results of the genetic algorithm optimized for the VO^{2+}/VO_2^+ electrolyte, evaluated for the reference network with mutation **combined with merging and splitting**, with: **(a)** the structure evolution over 1000 generations with the flow in the y-direction and the thickness in the z-direction with the membrane facing to the top, **(b)** the maximum fitness evolution, **(c)** the maximum electrical power and pumping power evolution, and **(d)** the polarization curve for the first and last generation.

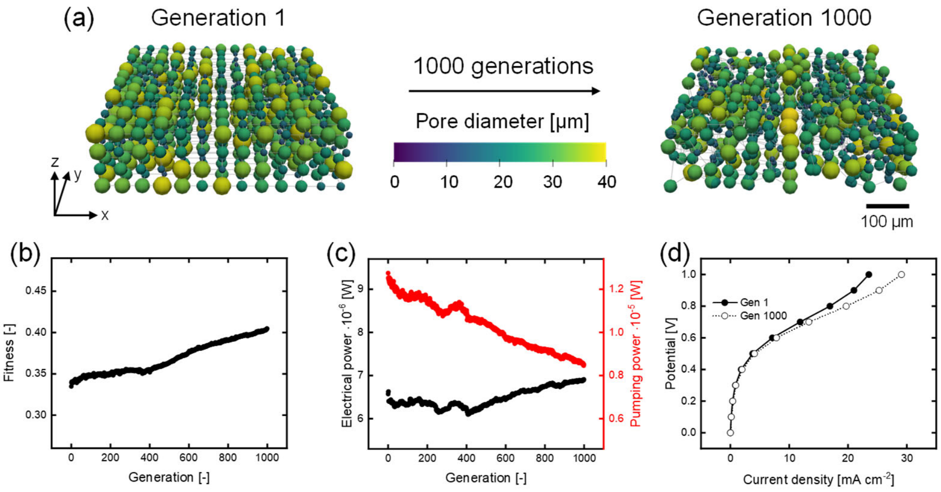

Figure A17: The results of the genetic algorithm optimized for the VO^{2+}/VO_2^+ electrolyte, evaluated for the reference network with **only merging and splitting**, with: **(a)** the structure evolution over 1000 generations with the flow in the y-direction and the thickness in the z-direction with the membrane facing to the top, **(b)** the maximum fitness evolution, **(c)** the maximum electrical power and pumping power evolution, and **(d)** the polarization curve for the first and last generation.

A5.2. The effect of merging and splitting

To study the influence of the *merging and splitting ratio*, simulations were performed with a 1:3 chance of merging over splitting (**Figure A18**) and a 2:3 chance (**Figure A19**), resulting in remarkable trends. First, both cases show improved fitness, increased electrochemical power, and decreased pumping power compared to the equal chance of merging and splitting, where the improvement in pumping power for both cases is even greater than for the reference system (i.e., the system with only mutation). Second, the 2:3 ratio results in a further enhancement of the pumping power because of the formation of more, and larger, pores. Third, the 1:3 ratio enhances the electrochemical performance by an increase in the internal surface area. Furthermore, compared to all other systems with merging and splitting, the mass transfer overpotential is reduced after optimization for the 1:3 ratio at 1 V, increasing the current output of the system. These observations align well with previous studies in the literature [4,10], where it is observed that many small pores enhance the electrochemical performance by providing more surface area and higher reaction rates. Moreover, the split pores are mainly formed near the membrane interface as most of the current is generated in this region [4,10]. Thus, the ratio between merging and splitting is shown to be an important parameter that can steer the electrode optimization toward the formation of networks with more or fewer pores than the starting network.

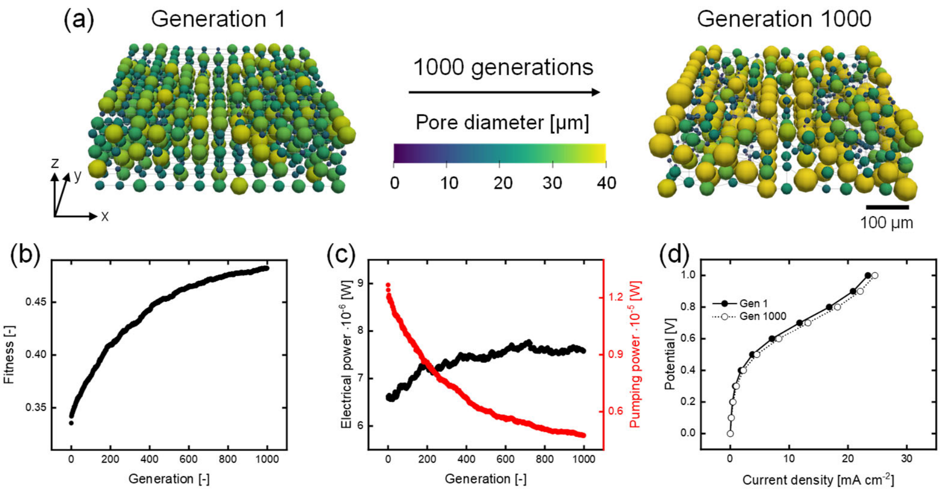

Figure A18: The results of the genetic algorithm optimized for the VO^{2+}/VO_2^+ electrolyte, evaluated for the reference network with mutation and merging and splitting with the **2:3 ratio**, with: **(a)** the structure evolution over 1000 generations with the flow in the y-direction and the thickness in the z-direction with the membrane facing to the top, **(b)** the maximum fitness evolution, **(c)** the maximum electrical power and pumping power evolution, and **(d)** the polarization curve for the first and last generation.

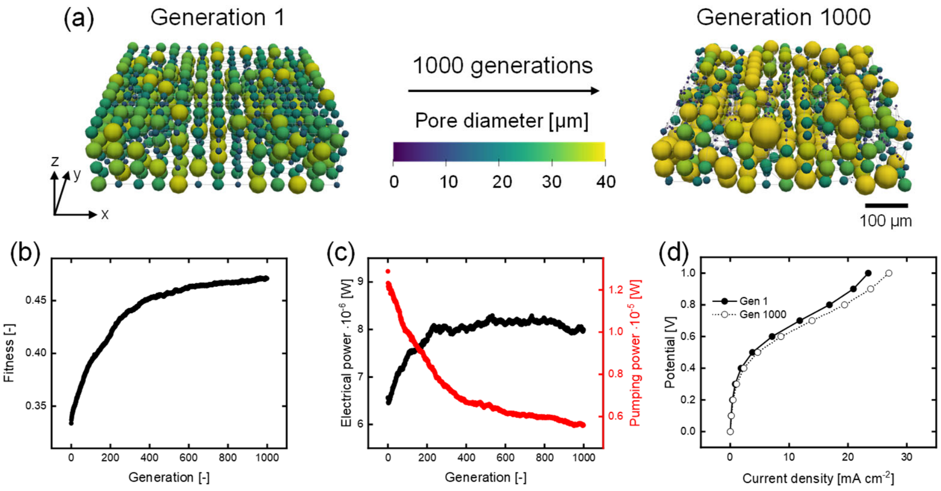

Figure A19: The results of the genetic algorithm optimized for the VO^{2+}/VO_2^+ electrolyte, evaluated for the reference network with mutation and merging and splitting with the **1:3 ratio**, with: **(a)** the structure evolution over 1000 generations with the flow in the y-direction and the thickness in the z-direction with the membrane facing to the top, **(b)** the maximum fitness evolution, **(c)** the maximum electrical power and pumping power evolution, and **(d)** the polarization curve for the first and last generation.

Table A6: The absolute values of the fitness and surface area for the best-performing network in the first and final generation for the mutation and merging and splitting evaluation.

	**Fitness [-]**		**Surface area [m²]**	
	**Gen 1**	**Gen 1000**	**Gen 1**	**Gen 1000**
Mutation	0.33	0.47	6.8×10^{-7}	9.0×10^{-7}
Mutation + Merging and Splitting	0.33	0.44	6.6×10^{-7}	7.2×10^{-7}
Only Merging and Splitting	0.33	0.40	6.7×10^{-7}	7.0×10^{-7}
No mutation	0.33	0.34	6.7×10^{-7}	6.5×10^{-7}
Mutation + dominated Merging (2:3)	0.34	0.48	6.8×10^{-7}	8.0×10^{-7}
Mutation + dominated Splitting (1:3)	0.33	0.47	6.7×10^{-7}	8.5×10^{-7}

Table A7: The absolute values of the electrical power and pumping power for the best-performing network in the first and final generation for the mutation and merging and splitting evaluation.

	**Electrical power [W]**		**Pumping power [W]**	
	**Gen 1**	**Gen 1000**	**Gen 1**	**Gen 1000**
Mutation	6.5×10^{-6}	8.5×10^{-6}	1.2×10^{-5}	5.9×10^{-6}
Mutation + Merging and Splitting	6.5×10^{-6}	7.0×10^{-6}	1.2×10^{-5}	6.6×10^{-6}
Only Merging and Splitting	6.5×10^{-6}	7.0×10^{-6}	1.3×10^{-5}	8.6×10^{-6}
No mutation	6.5×10^{-6}	6.5×10^{-6}	1.3×10^{-5}	1.2×10^{-5}
Mutation + dominated Merging (2:3)	6.5×10^{-6}	7.5×10^{-6}	1.2×10^{-5}	4.8×10^{-6}
Mutation + dominated Splitting (1:3)	6.5×10^{-6}	8.0×10^{-6}	1.3×10^{-5}	5.9×10^{-6}

Table A8: Percentual increase in fitness, electrical power, and pumping power over 1000 generations for the best-performing network in all evaluated systems for the mutation and merging and splitting evaluation.

**Percentual increase [%]**	**Fitness**	**Electrical power**	**Pumping power**	**Surface area**
Mutation	42	30	-54	34
Mutation + Merging and Splitting	31	6	-48	10
Only Merging and Splitting	21	5	-33	4
No mutation	4.3	-1.7	-5.0	-1.9
Mutation + dominated Merging (2:3)	44	15	-62	19
Mutation + dominated Splitting (1:3)	41	22	-57	27

A6. Network evolution

A6.1. Extracted network

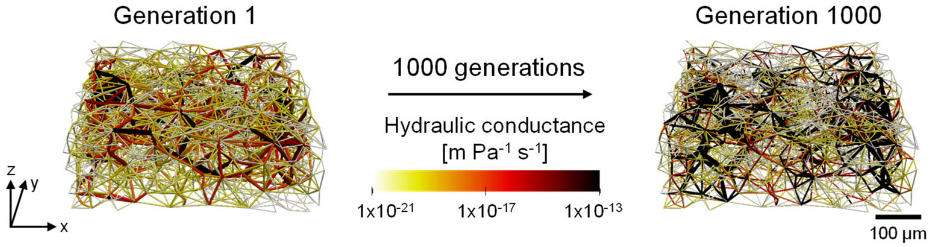

Figure A20: The hydraulic conductance of the throats for the first and last generation optimized for the VO^{2+}/VO_2^+ electrolyte, evaluated for the extracted Freudenberg H23 electrode (with mutation and a flow-through flow field) with surface area definition 2, with the flow in the y-direction and the thickness in the z-direction with the membrane facing to the top.

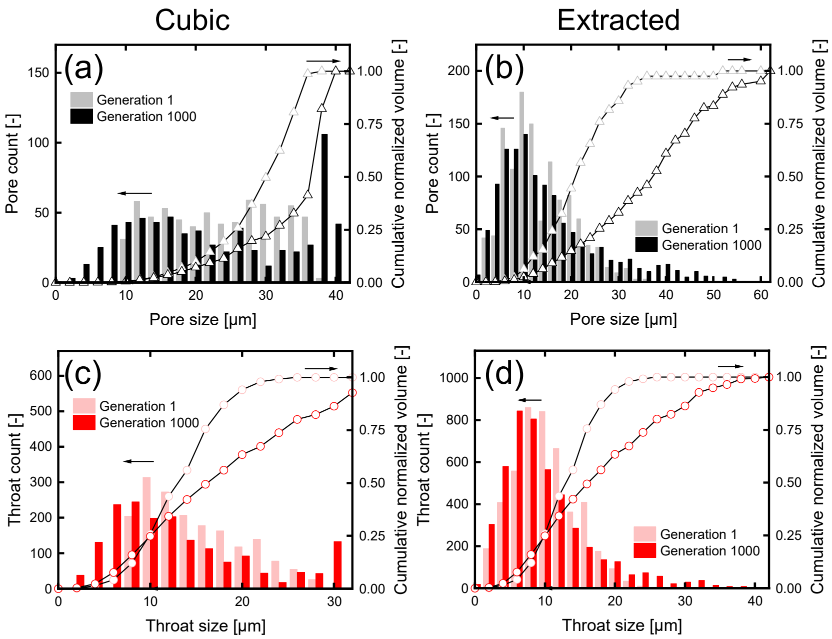

Figure A21: The **(a-b)** pore and **(c-d)** throat size distributions of the first and final generation, showing the pore or throat count and cumulative normalized volume distributions (divided in 2 μm pore or throat sized bins) for **(a)** the cubic network, and **(b)** the extracted network.

A6.2. Voronoi artificially generated network

The *Voronoi* network shows a strong optimization in the first ~70 generations, after which a local maximum is obtained followed by a decrease in the fitness value. Therefore, the optimization was only performed for 200 generations. The decrease in fitness for the Voronoi shows one disadvantage of using GAs as, when appropriate optimization conditions cannot be found, optimal solutions are not obtained [9].

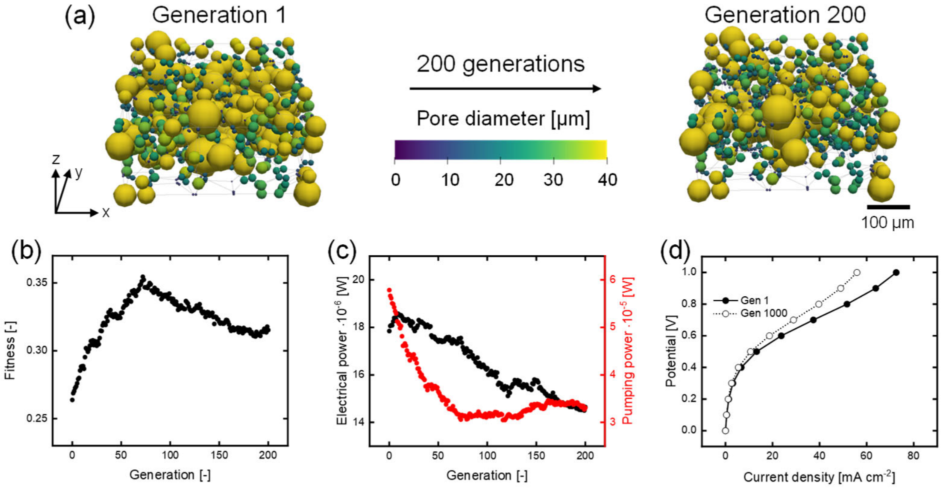

Figure A22: The results of the genetic algorithm optimized for the VO^{2+}/VO_2^+ electrolyte, evaluated for the **Voronoi network** (with mutation and a flow-through flow field) with surface area definition 2, with: **(a)** the structure evolution over 1000 generations with the flow in the y-direction and the thickness in the z-direction with the membrane facing to the top, **(b)** the maximum fitness evolution, **(c)** the maximum electrical power and pumping power evolution, and **(d)** the polarization curve for the first and last generation.

A7. Impact of the flow field design

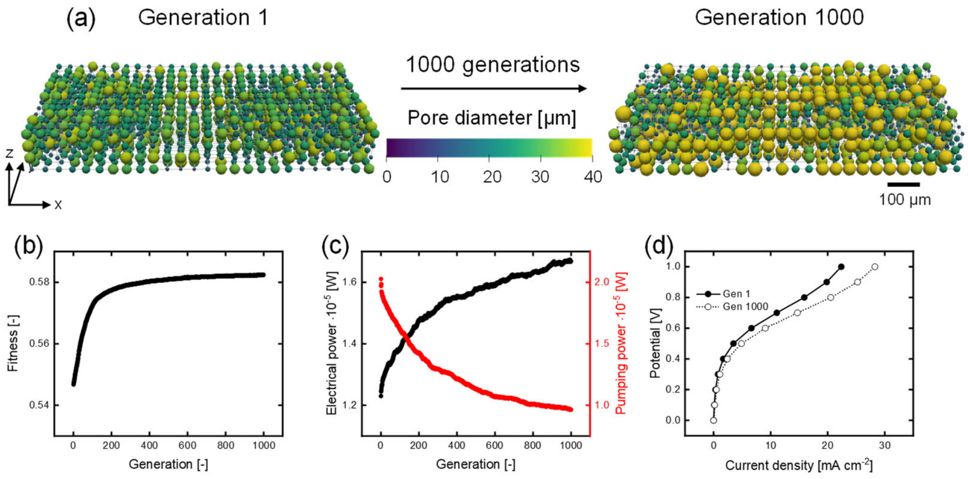

Figure A23: The results of the genetic algorithm optimized for the VO^{2+}/VO_2^+ electrolyte, evaluated for the reference network with the **interdigitated flow field**, with: **(a)** the structure evolution over 1000 generations with the flow in the y-direction and the thickness in the z-direction with the membrane facing to the top, **(b)** the maximum fitness evolution, **(c)** the maximum electrical power and pumping power evolution, and **(d)** the polarization curve for the first and last generation.

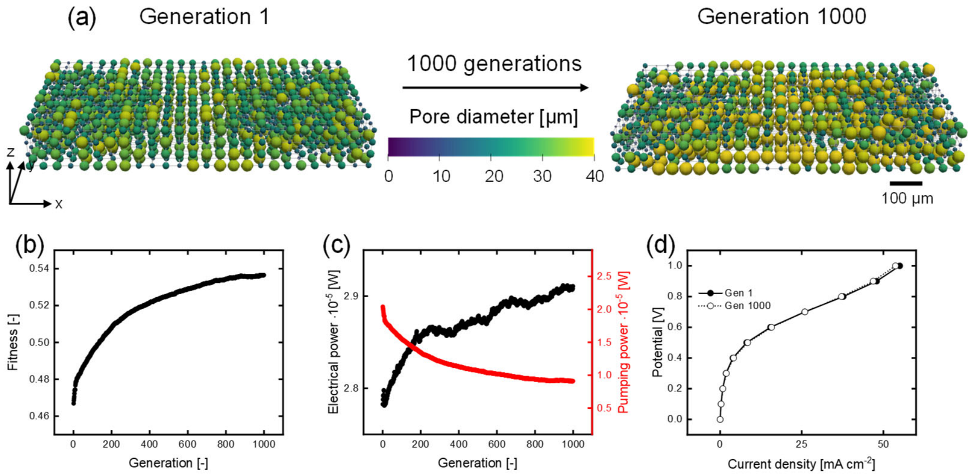

Figure A24: The results of the genetic algorithm optimized for the VO^{2+}/VO_2^+ electrolyte, evaluated for the reference network with the interdigitated flow field and with surface area **definition 2**, with: **(a)** the structure evolution over 1000 generations with the flow in the y-direction and the thickness in the z-direction with the membrane facing to the top, **(b)** the maximum fitness evolution, **(c)** the maximum electrical power and pumping power evolution, and **(d)** the polarization curve for the first and last generation.

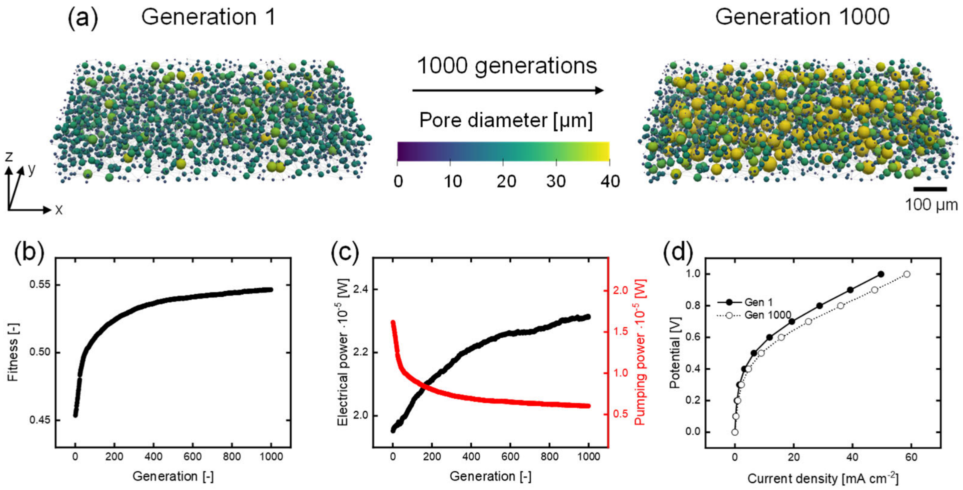

Figure A25: The results of the genetic algorithm optimized for the VO^{2+}/VO_2^+ electrolyte, evaluated for the **extracted Freudenberg H23 electrode** with the interdigitated flow field and with surface area definition 2, with: **(a)** the structure evolution over 1000 generations with the flow in the y-direction and the thickness in the z-direction with the membrane facing to the top, **(b)** the maximum fitness evolution, **(c)** the maximum electrical power and pumping power evolution, and **(d)** the polarization curve for the first and last generation.

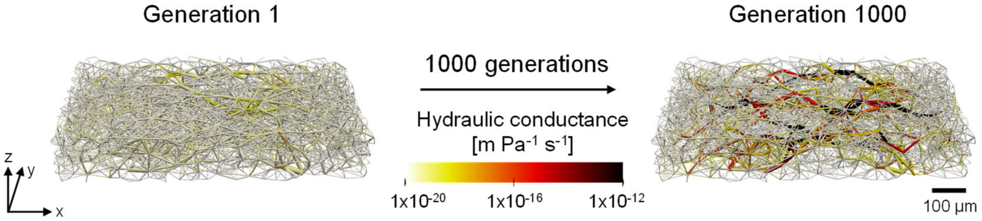

Figure A26: The hydraulic conductance of the throats for the first and last generation optimized for the VO^{2+}/VO_2^+ electrolyte, evaluated for the extracted Freudenberg H23 electrode and the interdigitated flow field (with mutation) with surface area definition 2, with the flow in the y-direction and the thickness in the z-direction with the membrane facing to the top.

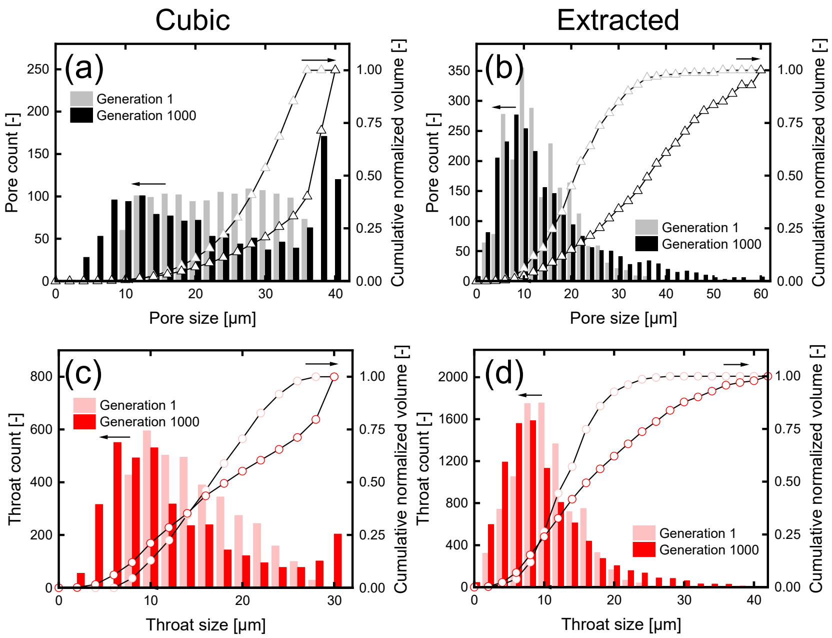

Figure A27: The **(a-b)** pore and **(c-d)** throat size distributions of the first and final generation, showing the pore or throat count and cumulative normalized volume distributions (divided in 2 µm pore or throat sized bins) for **(a)** the cubic network, and **(b)** the extracted network, both with the interdigitated flow field.

Table A9: The absolute values of the fitness and surface area for the best-performing network in the first and final generation for the initial structure and flow field design comparison. *All simulations were performed for 1000 generations with the exception of the Voronoi network (200 generations).

	**Fitness [-]**		**Surface area [m^2]**	
	**Gen 1**	**Gen 1000 */200**	**Gen 1**	**Gen 1000 */200**
Surface area definition 1				
Flow-through flow field Cubic	0.33	0.47	6.8 x 10^{-7}	9.0 x 10^{-7}
Interdigitated flow field Cubic	0.36	0.49	1.3 x 10^{-6}	1.7 x 10^{-6}
Surface area definition 2				
Flow-through flow field Cubic	0.44	0.53	1.4 x 10^{-6}	1.5 x 10^{-6}
Interdigitated flow field Cubic	0.47	0.54	2.8 x 10^{-6}	3.0 x 10^{-6}
Flow-through flow field Extracted	0.31	0.48	1.1 x 10^{-6}	1.4 x 10^{-6}
Interdigitated flow field Extracted	0.37	0.54	1.8 x 10^{-6}	2.7 x 10^{-6}
Flow-through flow field Voronoi	0.26	0.32	2.0 x 10^{-6}	1.6 x 10^{-6}

Table A10: The absolute values of the electrical power and pumping power for the best-performing network in the first and final generation for the initial structure and flow field design comparison. *All simulations were performed for 1000 generations with the exception of the Voronoi network (200 generations).

	Electrical power [W]		Pumping power [W]	
	**Gen 1**	**Gen 1000 */200**	**Gen 1**	**Gen 1000 */200**
Surface area definition 1				
Flow-through flow field Cubic	6.5×10^{-6}	8.5×10^{-6}	1.2×10^{-5}	5.8×10^{-6}
Interdigitated flow field Cubic	1.2×10^{-5}	1.7×10^{-5}	2.0×10^{-5}	9.6×10^{-6}
Surface area definition 2				
Flow-through flow field Cubic	1.4×10^{-5}	1.5×10^{-5}	1.2×10^{-5}	5.2×10^{-6}
Interdigitated flow field Cubic	2.8×10^{-5}	2.9×10^{-5}	2.0×10^{-5}	9.0×10^{-6}
Flow-through flow field Extracted	1.0×10^{-5}	1.2×10^{-5}	2.2×10^{-5}	7.8×10^{-6}
Interdigitated flow field Extracted	1.7×10^{-5}	2.3×10^{-5}	2.6×10^{-5}	6.0×10^{-6}
Flow-through flow field Voronoi	1.8×10^{-5}	1.5×10^{-5}	5.8×10^{-5}	3.3×10^{-5}

Table A11: Percentual increase in fitness, electrical power, and pumping power over 1000/200 generations for the best-performing network in all evaluated systems for the initial structure and flow field design comparison. *All simulations were performed for 1000 generations with the exception of the Voronoi network (200 generations).

**Percentual increase [%]**	**Fitness**	**Electrical power**	**Pumping power**	**Surface area**
Surface area definition 1				
Flow-through flow field Cubic	42	30	-54	34
Interdigitated flow field Cubic	37	36	-52	39
Surface area definition 2				
Flow-through flow field Cubic	19	3.6	-59	5.6
Interdigitated flow field Cubic	15	4.6	-55	6.4
Flow-through flow field Extracted	53	22	-65	31
Interdigitated flow field Extracted	48	39	-77	48
Flow-through flow field Voronoi	20	-19	-43	-16

Table A12: Total pore surface area values for each network layer for the best-performing network in the first and final generation from the current collector to the membrane, evaluated for the initial structure and flow field design comparison.

**Pore surface area x 10^{-7} [m^2]**	**Generation**	**Current collector**	**Internal 1**	**Internal 2**	**Membrane**
Flow-through flow field Cubic	1	1.9	1.5	1.3	1.8
	1000	2.5	1.8	2.2	2.4
Interdigitated flow field Cubic	1	3.5	2.6	2.8	3.1
	1000	5.1	4.0	3.6	4.6

A8. The effect of the electrolyte chemistry

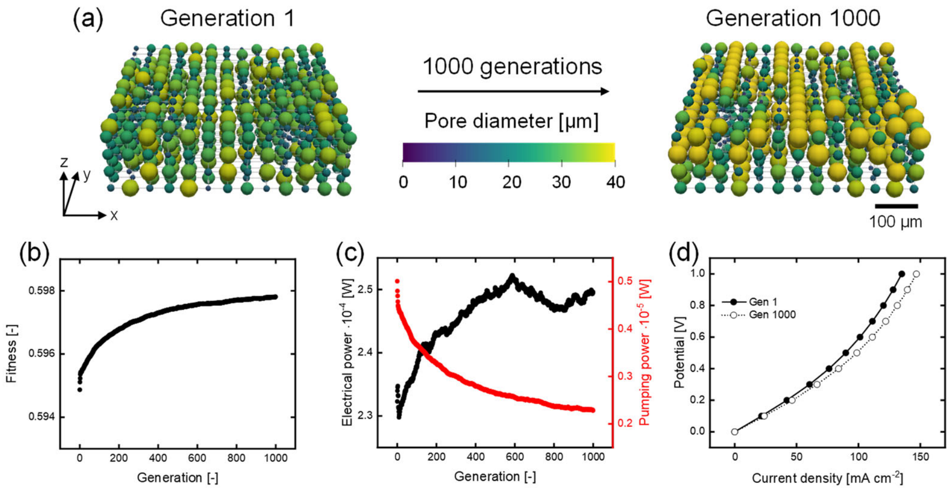

Figure A28: The results of the genetic algorithm optimized for the **TEMPO/TEMPO$^+$ electrolyte**, evaluated for the reference system (cubic network with mutation and a flow-through flow field), with: **(a)** the structure evolution over 1000 generations with the flow in the y-direction and the thickness in the z-direction with the membrane facing to the top, **(b)** the maximum fitness evolution, **(c)** the maximum electrical power and pumping power evolution, and **(d)** the polarization curve for the first and last generation.

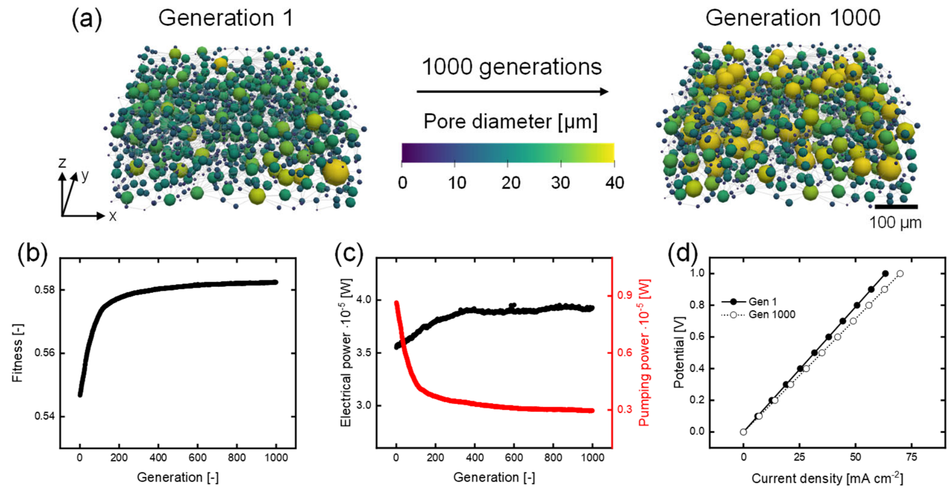

Figure A29: The results of the genetic algorithm optimized for the TEMPO/TEMPO$^+$ electrolyte, evaluated for the **extracted Freudenberg H23 electrode** (with mutation and a flow-through flow field) with surface area definition 2, with: **(a)** the structure evolution over 1000 generations with the flow in the y-direction and the thickness in the z-direction with the membrane facing to the top, **(b)** the maximum fitness evolution, **(c)** the maximum electrical power and pumping power evolution, and **(d)** the polarization curve for the first and last generation.

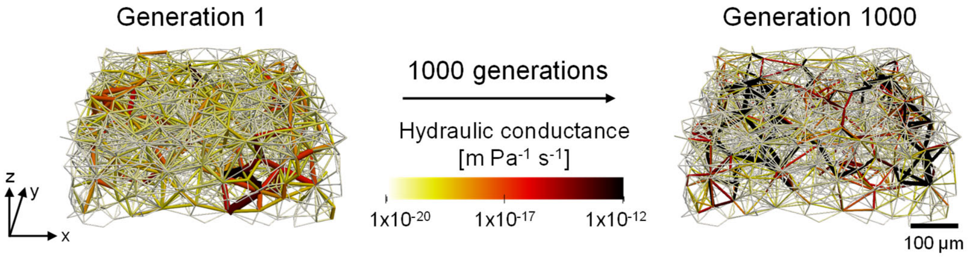

Figure A30: The hydraulic conductance of the throats for the first and last generation optimized for the TEMPO/TEMPO$^+$ electrolyte, evaluated for the extracted Freudenberg H23 electrode (with mutation and a flow-through flow field) with surface area definition 2, with the flow in the y-direction and the thickness in the z-direction with the membrane facing to the top.

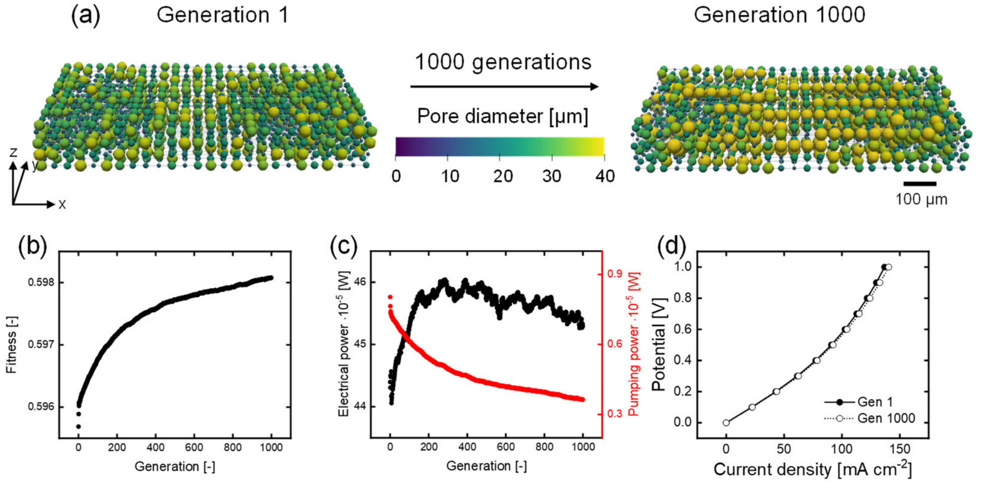

Figure A31: The results of the genetic algorithm optimized for the TEMPO/TEMPO$^+$ electrolyte, evaluated for the cubic network (with mutation) and an **interdigitated flow field**, with: **(a)** the structure evolution over 1000 generations with the flow in the y-direction and the thickness in the z-direction with the membrane facing to the top, **(b)** the maximum fitness evolution, **(c)** the maximum electrical power and pumping power evolution, and **(d)** the polarization curve for the first and last generation.

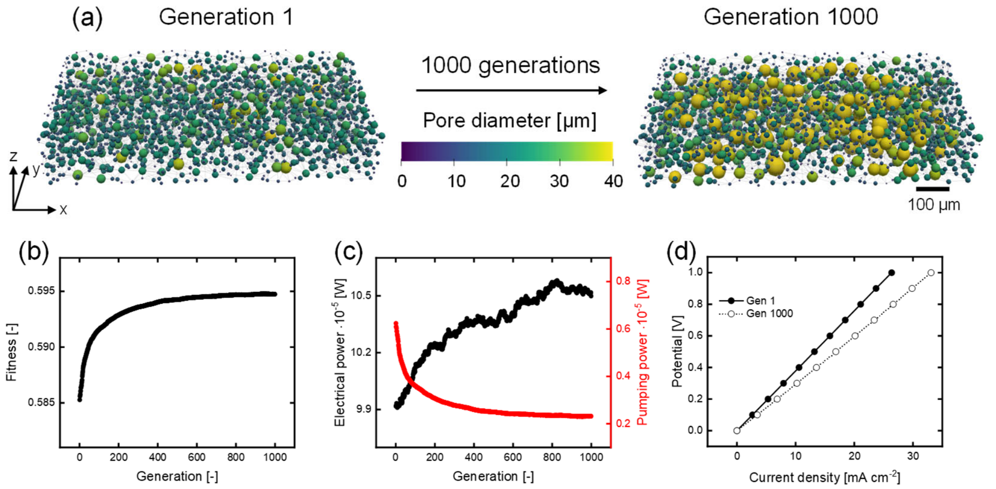

Figure A32: The results of the genetic algorithm optimized for the TEMPO/TEMPO$^+$ electrolyte evaluated for the **extracted Freudenberg H23 electrode** (with mutation) and an interdigitated flow field with surface area definition 2, with: **(a)** the structure evolution over 1000 generations with the flow in the y-direction and the thickness in the z-direction with the membrane facing to the top, **(b)** the maximum fitness evolution, **(c)** the maximum electrical power and pumping power evolution, and **(d)** the polarization curve for the first and last generation.

Table A13: The absolute values of the fitness and surface area for the best-performing network in the first and final generation for the chemistry comparison.

	**Fitness [-]**		**Surface area [m²]**	
	**Gen 1**	**Gen 1000**	**Gen 1**	**Gen 1000**
Surface area definition 1				
Vanadium Flow-through Cubic	0.33	0.47	6.8 x 10^{-7}	9.0 x 10^{-7}
Vanadium Interdigitated Cubic	0.36	0.49	1.3 x 10^{-6}	1.7 x 10^{-6}
TEMPO Flow-through Cubic	0.59	0.60	6.7 x 10^{-7}	7.8 x 10^{-7}
TEMPO Interdigitated Cubic	0.60	0.60	1.3 x 10^{-6}	1.5 x 10^{-6}
Surface area definition 2				
Vanadium Flow-through Extracted	0.31	0.48	1.1 x 10^{-6}	1.4 x 10^{-6}
TEMPO Flow-through Extracted	0.55	0.58	1.1 x 10^{-6}	1.4 x 10^{-6}
Vanadium Interdigitated Extracted	0.37	0.54	1.8 x 10^{-6}	2.7 x 10^{-6}
TEMPO Interdigitated Extracted	0.57	0.59	1.8 x 10^{-6}	2.6 x 10^{-6}

Table A14: The absolute values of the electrical power and pumping power for the best-performing network in the first and final generation for the chemistry comparison.

	**Electrical power [W]**		**Pumping power [W]**	
	**Gen 1**	**Gen 1000**	**Gen 1**	**Gen 1000**
Surface area definition 1				
Vanadium Flow-through Cubic	6.5 x 10^{-6}	8.5 x 10^{-6}	1.2 x 10^{-5}	5.8 x 10^{-6}
Vanadium Interdigitated Cubic	1.2 x 10^{-5}	1.7 x 10^{-5}	2.0 x 10^{-5}	9.6 x 10^{-6}
TEMPO Flow-through Cubic	2.3 x 10^{-4}	2.5 x 10^{-4}	5.0 x 10^{-6}	2.3 x 10^{-6}
TEMPO Interdigitated Cubic	4.4 x 10^{-4}	4.5 x 10^{-4}	8.0 x 10^{-6}	3.6 x 10^{-6}
Surface area definition 2				
Vanadium Flow-through Extracted	1.0 x 10^{-5}	1.2 x 10^{-5}	2.2 x 10^{-5}	7.8 x 10^{-6}
TEMPO Flow-through Extracted	3.6 x 10^{-5}	3.9 x 10^{-5}	8.6 x 10^{-6}	3.0 x 10^{-7}
Vanadium Interdigitated Extracted	1.7 x 10^{-5}	2.3 x 10^{-5}	2.6 x 10^{-5}	6.0 x 10^{-6}
TEMPO Interdigitated Extracted	7.6 x 10^{-5}	1.1 x 10^{-4}	1.0 x 10^{-5}	2.3 x 10^{-6}

Table A15: Percentual increase in fitness, electrical power, and pumping power over 1000 generations for the best-performing network in all evaluated systems for the chemistry comparison.

Percentual increase [%]	Fitness	Electrical power	Pumping power	Surface area
Surface area definition 1				
Vanadium Flow-through Cubic	42	30	-54	34
Vanadium Interdigitated Cubic	37	36	-52	39
TEMPO Flow-through Cubic	0.50	7.5	-54	18
TEMPO Interdigitated Cubic	0.40	2.0	-55	18
Surface area definition 2				
Vanadium Flow-through Extracted	53	22	-65	31
TEMPO Flow-through Extracted	6.5	11	-66	25
Vanadium Interdigitated Extracted	48	39	-77	48
TEMPO Interdigitated Extracted	4.5	39	-77	40

Table A16: Total pore surface area values for each network layer for the best-performing network in the first and final generation from the current collector to the membrane, evaluated for the chemistry comparison.

Pore surface area x 10^{-7} [m^2]	Generation	Current collector	Internal 1	Internal 2	Membrane
Vanadium Flow-through Cubic	1	1.9	1.5	1.3	1.8
	1000	2.5	1.8	2.2	2.4
TEMPO Flow-through Cubic	1	1.8	1.3	1.4	1.8
	1000	1.6	1.7	2.2	2.4

A9. Simulation parameters

The parameters used for the simulations described in this work can be found in **Table A17**. These can be set in the "GA_main" script available at the following online repository: https://github.com/MaximevdHeijden/GA-RFB-electrode/.

Table A17: The parameters set in the model to run all the performed simulations presented in this work. [1]Surface area definition as mentioned in **Section A4.1**. The surface area definition can be changed in "openpnm/models/geometry/pore_surface_area". [2]The fitness function has been changed according to **Section A4.4**. The fitness function can be altered in the GA_Functions script in the "determine_fitness" function. [3]The chemistry can be altered, for which another inputdict should be selected and changed in the "GA_main" and "customFunctionsGA" scripts.

Simulation	Network shape	Network type	Flow field	Mutation	M&S	Throat factor	M&S ratio
**Reference**	13x14x4	Cubic	FTFF	Yes	No	0.8	-
**SA definition 2**[1]	13x14x4	Cubic	FTFF	Yes	No	0.8	-
**SA definition 3**[1]	13x14x4	Cubic	FTFF	Yes	No	0.8	-
**SA definition 4**[1]	13x14x4	Cubic	FTFF	Yes	No	0.8	-
**SA definition 1 extracted**[1]	-	Extracted	FTFF	Yes	No	0.8	-
**SA definition 2 extracted**[1]	-	Extracted	FTFF	Yes	No	0.8	-
**Troat factor 0.7**	13x14x4	Cubic	FTFF	Yes	No	0.7	-
**Throat factor 0.9**	13x14x4	Cubic	FTFF	Yes	No	0.9	-
**400 µm in thickness**	13x14x8	Cubic	FTFF	Yes	No	0.8	-
**Pumping power**[2]	13x14x4	Cubic	FTFF	Yes	No	0.8	-
**Electrochemical power**[2]	13x14x4	Cubic	FTFF	Yes	No	0.8	-
**Merging and Splitting**	13x14x4	Cubic	FTFF	Yes	Yes	0.8	0.5
**Only Merging and Splitting**	13x14x4	Cubic	FTFF	No	Yes	0.8	0.5
**No mutation**	13x14x4	Cubic	FTFF	No	No	0.8	-
**Dominated Merging**	13x14x4	Cubic	FTFF	Yes	Yes	0.25	0.5
**Dominated Splitting**	13x14x4	Cubic	FTFF	Yes	Yes	0.75	0.5
**IDFF Cubic**	13x14x4	Cubic	IDFF	Yes	No	0.8	-
**IDFF SA definition 2**[1]	13x14x4	Cubic	IDFF	Yes	No	0.8	-
**IDFF Extracted**	-	Extracted	IDFF	Yes	No	0.8	-
**Voronoi**	-	Voronoi	FTFF	Yes	No	0.8	-
**TEMPO**[3]	13x14x4	Cubic	FTFF	Yes	No	0.8	-
**TEMPO IDFF**[3]	13x14x4	Cubic	IDFF	Yes	No	0.8	-
**TEMPO extracted**[3]	-	Extracted	FTFF	Yes	No	0.8	-
**TEMPO IDFF extracted**[3]	-	Extracted	IDFF	Yes	No	0.8	-

A10. References

[1] M. A. Sadeghi, M. Aganou, M. Kok, M. Aghighi, G. Merle, J. Barralet, J. Gostick, *J. Electrochem. Soc.* **166**, A2121–A2130 (2019).

[2] C. T. Wan, R. R. Jacquemond, Y. Chiang, K. Nijmeijer, F. R. Brushett, A. Forner-cuenca, *Adv. Mater.* **33**, 2006716 (2021).

[3] A. Forner-Cuenca, E. E. Penn, A. M. Oliveira, F. R. Brushett, *J. Electrochem. Soc.* **166**, A2230–A2241 (2019).

[4] M. van der Heijden, R. van Gorp, M. A. Sadeghi, J. Gostick, A. Forner-Cuenca, *J. Electrochem. Soc.* **169**, 040505 (2022).

[5] R. van Gorp, M. van der Heijden, M. A. Sadeghi, J. Gostick, A. Forner-Cuenca, *Chem. Eng. J.* **455**, 139947 (2023).

[6] J. T. Gostick, *Phys. Rev. E.* **96**, 1–15 (2017).

[7] R. R. Jacquemond, C. T.-C. Wan, Y.-M. Chiang, Z. Borneman, F. R. Brushett, K. Nijmeijer, A. Forner-Cuenca, *Cell Reports Phys. Sci.* **3**, 100943 (2022).

[8] V. Muñoz-Perales, M. van der Heijden, V. de Haas, J. Olinga, M. Vera, A. Forner-Cuenca, *ChemElectroChem*. **11**, e2023003 (2024).

[9] K. Thoiba Meetei, *Int. J. Sci. Res.* **3**, 231–235 (2014).

[10] C. T. C. Wan, R. R. Jacquemond, Y. M. Chiang, A. Forner-Cuenca, F. R. Brushett, *Energy Technol.* **11**, 2300137 (2023).
